# Oral and fecal microbiome of confiscated Bengal slow lorises in response to confinement duration

**DOI:** 10.3389/fmicb.2022.941261

**Published:** 2022-09-27

**Authors:** Qingyong Ni, Shasha Dong, Bolin Xing, Bo Zeng, Fanli Kong, Huailiang Xu, Yongfang Yao, Diyan Li, Mingwang Zhang, Xiaolan Fan, Deying Yang, Mingyao Yang, Meng Xie

**Affiliations:** ^1^Key Laboratory of Livestock and Poultry Multi-omics, Ministry of Agriculture and Rural Affairs, College of Animal Science and Technology, Sichuan Agricultural University, Chengdu, China; ^2^Farm Animal Genetic Resources Exploration and Innovation Key Laboratory of Sichuan Province, Sichuan Agricultural University, Chengdu, China; ^3^College of Life Science, Sichuan Agricultural University, Yaan, China

**Keywords:** *Nycticebus bengalensis*, microbiome, captivity, pathogens, husbandry management, reintroduction

## Abstract

Slow lorises are small arboreal and nocturnal primates. Due to the illegal trade, a large number of slow lorises were confiscated into wildlife sanctuaries or rescue centers. The re-release has been considered a preferable approach for alleviating the captive pressure, but inappropriate and long-term confinement make it difficult to achieve this goal. In this study, we investigated and compared the fecal and oral microbiome of Bengal slow lorises (*Nycticebus bengalensis*) under long-term captivity (LC) and short-term captivity (SC) groups based on 16s rRNA high-throughput gene sequencing. The oral microbiome displayed higher Chao1 richness but lower Shannon and Simpson indices than the fecal microbiome. The Bengal slow lorises under long-term captivity had abundant pathogenic genera in both gut and oral microbiomes, such as *Desulfovibrio*, *Actinomyces*, *Capnocytophaga*, *Neisseria*, and *Fusobacterium*, while some specific bacterial taxa associated with intestinal balance were more enriched in the SC group. Due to the plant gum scarcity in the diet, both groups had a low abundance of *Bifidobacterium*. Function profile prediction indicated that the LC group was enriched with genetic information processing and metabolism pathways due to the stable food intake. The increased membrane transport and xenobiotic metabolism and degradation functions in the SC group could be explained by the function of the host microbiome in facilitating adaptation to changing environments and diets. The results demonstrated that the oral microbiome had the potential to be used as a regular surveillance tool. Also, current captive management should be improved to ensure reintroduction success.

## Introduction

Human-constructed environments represent extreme changes from the natural conditions, including restrictions in diet, reduction of range and habitat types, antibiotics and other veterinary interventions, altered intraspecific interactions, and increased exposure to humans for the wild animals kept in captivity (McKenzie et al., 2017; Dallas and Warne, 2022). These lifestyle disruptions significantly impact wildlife physiology and health status, which are linked with their wellbeing. These effects are important to improve the conservation outcomes for the endangered animal species (Jin Song et al., 2019; Trevelline et al., 2019). The animal can be regarded as a multispecies hybrid organism composed of host and microbes, and the microbial communities can be drastically altered by a variety of factors, including diet, environments, medical interventions, and disease states. The microbiome plays a vital role in host nutrition, metabolism, immunity, development, and behavior. Microbiomics can determine the host-environment interactions, help develop predictive biomarkers for certain diseases, and confront longstanding health issues (Barko et al., 2018).

Most microbiome studies focus on the gastrointestinal tract. Although the microbes inhabit multiple body parts, and fecal samples are generally used to represent the gut microbiota (Clayton et al., 2018b). Captivity alters the availability and diversity of food resources and reduces gut microbiota abundance and diversity (Borbón-García et al., 2017). The reduction of diversity in gut microbiota may imply the loss or decrease of microbial functional groups. The microbiome may be less efficient, less resilient, and more susceptible to pathogens (Peixoto et al., 2021). Clayton et al. (2016) found that the severity of captivity was associated with the reduced gut microbial diversity and the disruption level to the native gut microbiota. The microbial composition of the gastrointestinal tract is sensitive to changes in diet and stressors of captive animals under artificial environments, which can lead to dysbiosis and disease (Rinninella et al., 2020). Captive animals have distinct gut microbiota, and individuals suffering from the disease have distinct microbial characteristics compared to healthy individuals (Amato et al., 2016; Tong et al., 2020).

In addition to the gut microbiome, the oral microbiome is considered a new target for evaluating host health and disease. Understanding the oral microbial divergences, the relative abundance and functional activity, as well as genetic factors and ecological pressures, is a primary focus of research concerning oral and body health (Wade, 2013). An increasing number of studies have reported that the oral microbiome is correlated with dental caries, endodontic infections, gingivitis, periodontitis, and other oral diseases (Costalonga and Herzberg, 2014; Sampaio-Maia et al., 2016; Ebersole et al., 2020). Furthermore, the oral and oropharyngeal microbiota can reach the stomach and spread through the body by swallowing saliva, nutrients, and drinks (Olsen and Yamazaki, 2019). The oral cavity may act as a reservoir of potential pathogens for pneumonia and cardiovascular diseases in both animals and humans (Blekkenhorst et al., 2018; Kitsios et al., 2018; Matsha et al., 2020). The oral microbiome may exhibit various patterns in different wild animal species compared to the gut microbiome due to the closer contact of saliva with the environment (Li et al., 2013). However, only a limited number of studies have explored the relationships between their captive issues and the oral microbiome (Hyde et al., 2016; Sawaswong et al., 2020, 2021).

The Asian slow lorises (Genus *Nycticebus*, family Lorisidae) are small arboreal and nocturnal primates. All the species are recognized as threatened (Vulnerable, Endangered, or Critically Endangered) by the International Union for Conservation of Nature (IUCN) Red List and listed in the Convention on International Trade in Endangered Species of Wild Fauna and Flora (CITES) (Nekaris, 2014). While the wild populations are rapidly declining in native habitats, many individuals are confiscated due to illegal trade and are kept in zoos or rescue centers in captive environments (Nekaris et al., 2010; Ni et al., 2018). The slow lorises are at particular risk of physical and behavioral harm from trade and captive practices due to their highly specific and complex husbandry requirements and their specific environmental and dietary niches (Fuller et al., 2018). Behavioral abnormalities and dental problems are common issues for the confiscated slow lorises, leading to physical and psychological trauma, which causes the mortality of captive animals (Fuller et al., 2014). In addition, slow lorises’ bites inflicted with the mixture of brachial gland exudate and their saliva can cause anaphylactic shock in humans and result in edema, fester and leave loss of fur and scarring in loris conspecifics, making them the only venomous primates (Nekaris et al., 2013). During the transport and captivity in illegal trade, the teeth of a large number of slow lorises are removed as a precaution against their bite (Nijman et al., 2017). Such injuries propose higher requests for sanctuary housing and welfare and make their reintroduction impossible (Fuller et al., 2018).

Bacterial diversity and composition are the indicators of reduced diet diversity and reduced contact with variable environmental substrates for captive animals (Kohl et al., 2014). Microbiome monitoring can improve the husbandry management of wild animals by rapidly identifying shifts in their microbiota (Hale et al., 2018), and analysis of microbial ecology is crucial for conservation practices of endangered animal species (Redford et al., 2012; Tang et al., 2020). Most previous studies examined how the microbial communities varied in association with the level of captivity by means of comparative analysis between wild and captive populations in different habitats (Gibson et al., 2019; Sun et al., 2019). Despite the emerging research interest in the effects of dietary improvement on the gut microbiota of slow lorises (Cabana et al., 2019; Ni et al., 2020), there is still limited knowledge of their microbial shifts during the rescuing process. Such data paucity in quantitative evaluation of husbandry effects on the confiscated animals has impeded the efforts to improve the success rate in subsequent reintroduction and rehabilitation based on the soft-release strategy. While the rescue centers released the confiscated slow lorises into the wild within a few months, the confinement duration was extended due to the outbreak of coronavirus disease 19 (COVID-19) in 2020 and 2021, making it possible to examine their physical or ecological responses to the duration of captivity. Thus, we conducted a comparative study on the oral and fecal microbiome of captive Bengal slow lorises (*Nycticebus bengalensis*) to determine the divergence of microbiota along the gastrointestinal tract for the individuals undergoing inappropriate confinement and correlate the variations with animal welfare and husbandry management.

## Materials and methods

### Animal ethics statement

Sample collection and animal experiments were performed by the Institutional Review Board (IRB13627) and the Institutional Animal Care and Use Committee of the Sichuan Agricultural University, China, under permit number DKY-2020302166, as well as the Administration for Wild Animal Protection in Yunnan Provinces, China and adhered to the American Society of Primatologists Principles for the Ethical Treatment of Non-Human Primates.

### Sample collection

The study was conducted at Dehong Wildlife Rescue Center in Yunnan, China (24.38287°N, 98.45872°E). A total of 85 Bengal slow lorises, confiscated from illegal trade or personal captivities, were separately housed in small iron cages (80 × 50 × 60 cm^3^), and the cages were grouped into two indoor enclosures. There are no breeding centers in surrounding regions. Thus, it is assumed that all the individuals are wild-caught. Thirty-seven individuals housed in the rescue center for more than 13 months (ranging from 13 to 18 months) were defined as group LC (long-term captivity). The individuals in another enclosure (*N* = 48) were confiscated within 12 months (ranging from 6 to 12 months) and were defined as SC (short-term captivity) group (Table 1). A physical examination was conducted by a veterinarian before sample collection. Compared with SC group, more individuals in the LC group had severe dental problems (Cross tabs with Chi-square test, *x*^2^ = 3.544, *P* = 0.060), mostly canine teeth loss. More individuals were disabled or received amputation (*x*^2^ = 4.973, *P* = 0.026). In addition, the LC group had lower body weight (Mann–Whitney *U* test, z = −3.905, *P* < 0.001) under the same dietary supply (50 g peeled bananas, 50 g apples, 40 g rice, and 10 g frozen locusts).

**TABLE 1:** Captivity-related information of the two slow loris groups.

Group		Long-term captivity (LC)	Short-term captivity (SC)
Confinement duration (months)		13–18	6–12
Number of individuals	Total	37	48
	Male/Female	24/13	17/31
	Dental problem	26 (70.3%)	24 (50.0%)
	Disabled	12 (32.4%)	6 (12.5%)
Bodyweight (g)		1005.4 ± 259.8	1232.3 ± 226.3
Alpha diversity indices	Chao1-fecal	663.73 ± 194.67	689.41 ± 130.68
	Chao1-oral	1187.65 ± 483.59	1318.58 ± 585.48
	Shannon-fecal	5.29 ± 0.68	5.40 ± 0.63
	Shannon-oral	5.18 ± 0.57	4.64 ± 0.95
	Simpson-fecal	0.92 ± 0.05	0.93 ± 0.03
	Simpson-oral	0.90 ± 0.04	0.83 ± 0.10

Fecal samples were collected from the trays placed under the cages. We cleared the trays at 1 a.m. and collected the feces at 6 a.m. to ensure that all the samples were collected within 5 h after defecation. We used a long-handle polyester-tipped swab (BKMAM, China) to reach into the cage for oral sample collection and collected oral samples from individuals one by one at night. We held the swab until they opened their mouth and then took it back immediately. The collected fecal and oral samples were maintained in dry ice, transferred to the laboratory within 12 h, and kept at −80°C for future use.

### Isolation and deoxyribonucleic acid sequencing

The total genome deoxyribonucleic acid (gDNA) was extracted from samples using the cetyl trimethylammonium bromide (CTAB) method (Stefanova et al., 2013). DNA concentration and purity were checked on 1% agarose gels, and DNA was diluted to 1 ng/μL using sterile water according to the concentration. The V4 region of the 16S rRNA gene was amplified based on primers 515F (5′-GTGCCAGCMGCCGCGGTAA-3′) and 806R (5′-GGACTACHVGGGTWTCTAAT-3′). PCR reaction for each sample was carried out with 15 μl of Phusion^®^ High-Fidelity PCR Master Mix (New England Biolabs, Ipswich, MA, United States), 2 μM of forward and reverse primers, and 10 ng of DNA template. The PCR conditions were as follows: 98°C for 1 min, 30 cycles at 98°C for 10 s, 50°C for 30 s, 72°C for 30 s, and 72°C for 5 min. PCR products were visualized by electrophoresis on 2% agarose gel and purified with Qiagen Gel Extraction Kit (Qiagen, Hilden, Germany). The sequencing libraries were generated using TruSeq^®^ DNA PCR-Free Sample Preparation Kit (Illumina, San Diego, CA, United States). The library quality was assessed on the Qubit@2.0 Fluorometer (Thermo Scientific, Waltham, United States) and Agilent Bioanalyzer 2100 system. The library was sequenced on an Illumina NovaSeq platform, and 250 bp paired-end reads were generated.

### Statistical analysis

The overlapping regions of pair-end reads were merged using FLASH (V1.2.7^1^) (Magoè and Salzberg, 2011), and the splicing sequences, namely, raw tags, were quality-filtered based on the QIIME (V1.9.1^2^) (Caporaso et al., 2010). The high-quality clean tags were compared with the reference database (Silva database^3^) (Quast et al., 2012) using the UCHIME algorithm (UCHIME^4^) (Edgar et al., 2011). The chimera sequences were detected and removed as described previously (Haas et al., 2011). Then, we obtained the effective tags. The sequence data were deposited in the National Genomics Data Center (NGDC)^5^ (Accession numbers: CRA008207 and CRA008208).

The Uparse (v7.0.1001^6^) was used to perform sequence analysis, and the sequences with ≥97% similarity were assigned to the same operational taxonomic units (OTUs). Singletons (OTUs represented by only one sequence) were filtered out from the resulting OTU table. The representative sequence for each OTU was screened, and the Silva Database was used to annotate taxonomic information based on the Mothur algorithm. Alpha diversity indices (Chao1 and Shannon) were calculated by QIIME from rarefied samples and displayed with R (Version 2.15.3). The estimators were compared by the Mann–Whitney *U* test. Beta diversity of the fecal samples between different groups was calculated using binary Jaccard, and weighted UniFrac was used for the oral samples. A one-way analysis of similarity (ANOSIM) was used to determine the differences in bacterial communities. Differential bacterial abundance comparison between LC and SC groups was investigated by linear discriminant analysis (LDA) effect size (LefSe) tool. A size-effect threshold of 3.0 on the logarithmic LDA score was used for selecting the significantly different taxa (*P* < 0.05). *P* values were adjusted using the Benjamini-Hochberg false discovery rate (FDR) method (Benjamini and Hochberg, 1995). Phylogenetic Investigation of Communities by Reconstruction of Unobserved States (PICRUSt) was used to predict the functional inference of microbiome (Langille et al., 2013), and the multiple *t*-test was used to analyze the significant difference between the groups (*P* < 0.05).

## Results

### Metadata and sequencing summary

We obtained 6,590,790 high-quality filtered reads from 77 fecal samples, corresponding to 78,976 ± 4,317 reads per sample, and got 7,218,194 sequences (mean 84,920 ± 5,262) from 85 oral samples, generating an average reading length of 253 bp (Supplementary Table 1). A total of 3,203 OTUs were obtained from the fecal samples at a sequence similarity level of 97%, while a total of 7,861 OTUs were obtained from the oral samples. The rarefaction curves showed that the number of observed OTUs increased with the sequencing depth, indicating the number of OTUs was sufficient for further analysis (Supplementary Figure 1). In addition, Good’s Coverage rate of each sample was close to 99% (Supplementary Table 2), indicating that most microbial species were detected.

### Diversity of fecal and oral microbiome profiles

**FIGURE 1:**
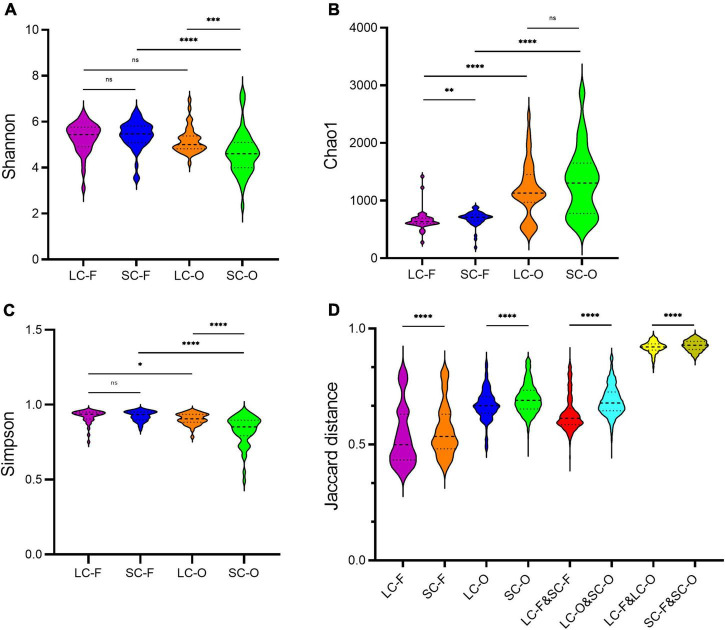
The alpha and beta diversity in the fecal-oral microbiome of slow loris groups under different confinement durations: long-term captivity-fecal (*LC-F*), short-term captivity-fecal (*SC-F*), long-term captivity-oral (*LC-O*), short-term captivity-oral (*SC-O*). **(A)** Shannon’s diversity index; **(B)** Chao1 index; **(C)** Simpson index; **(D)** the Jaccard distances of the beta diversity between communities. The differences were tested by Mann–Whitney *U* test (*ns*, not significant, **P < 0.01, ***P < 0.001, ****P < 0.0001).

The oral microbiome in the LC group had higher Shannon (Mann–Whitney *U* test, *P* < 0.001) and Simpson (*P* < 0.001) indices compared to the SC group (Table 1 and Figures 1A,C). However, the Chao1 diversity of the fecal microbiome was significantly lower (*P* = 0.003) in the LC group than that in the SC group (Figure 1B). The Shannon (*P* = 0.537) and Simpson (*P* = 0.858) indices of fecal microbiota and the Chao1 diversity in oral samples (*P* = 0.263) showed no significant differences between the groups. In both SC and LC groups, the oral microbiome exhibited significantly higher Chao1 richness (*P* < 0.001) and lower Simpson index (*P* = 0.013) than the fecal microbiome. The Shannon index was not significantly different between oral and fecal samples of the LC group (*P* = 0.092). It was significantly lower (*P* < 0.001) in the oral microbiome of SC than in the fecal microbiome.

**FIGURE 2:**
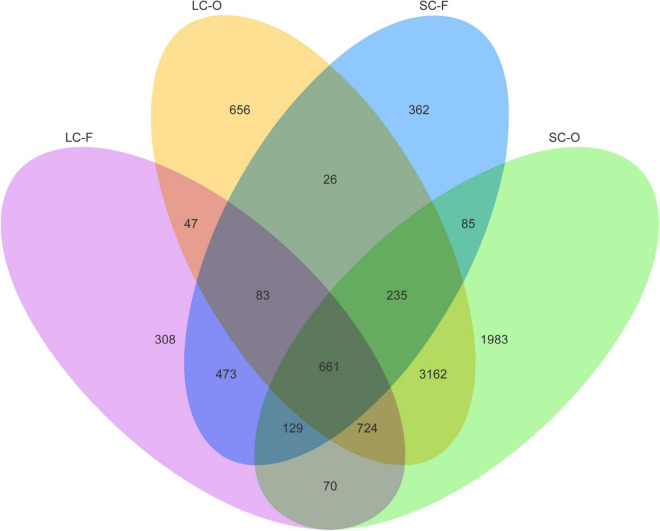
Venn diagram showing the number of shared operational taxonomic units (OTUs) identified from each group.

**FIGURE 3:**
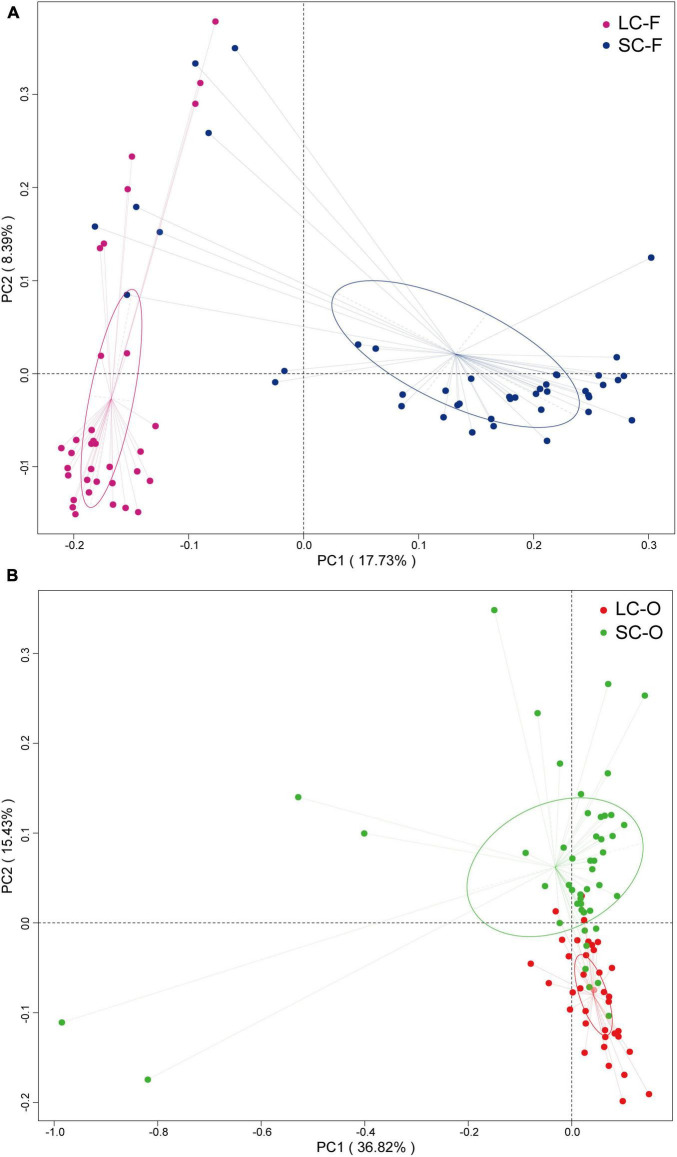
Principal coordinate analysis (PCoA) analysis of gut microbiota based on binary Jaccard distances **(A)** and oral microbiota based on weighted UniFrac dissimilarities **(B)**.

According to the Venn diagram, 661 OTUs were shared by all groups (Figure 2). A large proportion of sharing OTUs (4782, 60.8%) was found between oral samples in SC and LC groups, while the fecal microbiome had fewer overlapped OTUs (1515, 47.3%) between the two groups. The oral microbiota of SC harbored the highest number of unique OTUs compared to other groups. We calculated the intra- and inter-group Jaccard distances and examined the differences by the Mann–Whitney *U* test. The SC group had higher community distances (*P* < 0.001) in both oral and fecal samples than those in the LC group (Figure 1D). A statistically higher distance (*P* < 0.001) was found in the oral microbiome between the two groups than in the fecal microbiome and the oral-fecal distance in SC group compared to the LC group. The principal coordinate analysis (PCoA) plots showed that fecal and oral samples were clustered by the group based on binary Jaccard (Figure 3A) and weighted UniFrac distance (Figure 3B) separately. ANOSIM results revealed significant differences in both fecal (*R* = 0.121, *P* = 0.001) and oral microbial communities (*R* = 0.170, *P* = 0.001) between LC and SC groups.

### Relative abundance and dominant taxa

**FIGURE 4:**
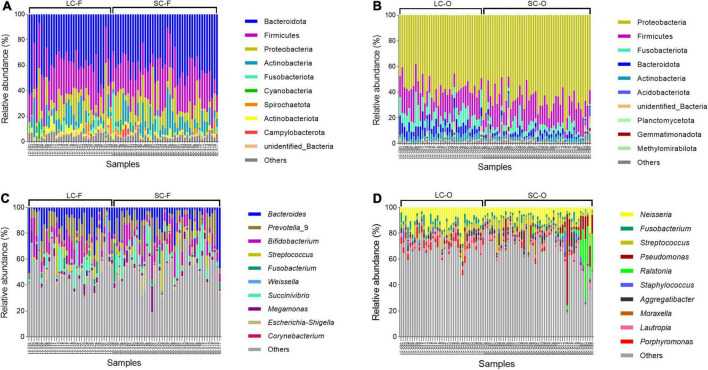
Relative abundance of the top 10 bacterial phylum and genera in the fecal **(A,C)** and oral samples **(B,D)**.

The dominant phyla in fecal microbiota of Bengal slow lorises were Bacteroidota (LC: 40.1 ± 13.3%; SC: 41.0 ± 11.3%), Firmicutes (LC: 26.8 ± 7.4%; SC: 26.1 ± 7.9%), and Proteobacteria (LC: 12.0 ± 11.5%; SC: 13.2 ± 9.7%) (Figure 4A). The oral samples were dominated by Proteobacteria (LC: 56.5 ± 7.2%; SC: 66.6 ± 9.1%), Firmicutes (LC: 18.5 ± 5.7%; SC: 17.9 ± 6.7%), and Fusobacteriota (LC: 9.5 ± 4.6%; SC: 4.8 ± 2.9%) (Figure 4C). At the genus level, the fecal microbiota were dominated by *Bacteroides* (LC: 12.5 ± 11.3%; SC: 10.5 ± 10.4%), *Prevotella*_9 (LC: 10.5 ± 7.8%; SC: 9.5 ± 8.3%), and *Bifidobacterium* (LC: 8.7 ± 7.4%; SC: 8.8 ± 6.6%) (Figure 4B), while *Neisseria* (LC: 11.0 ± 5.6%; SC: 6.7 ± 3.7%), *Fusobacterium* (LC: 56.5 ± 7.2%; SC: 66.6 ± 9.1%), and *Streptococcus* (LC: 56.5 ± 7.2%; SC: 66.6 ± 9.1%) had higher relative abundances in oral microbiota (Figure 4D).

**FIGURE 5:**
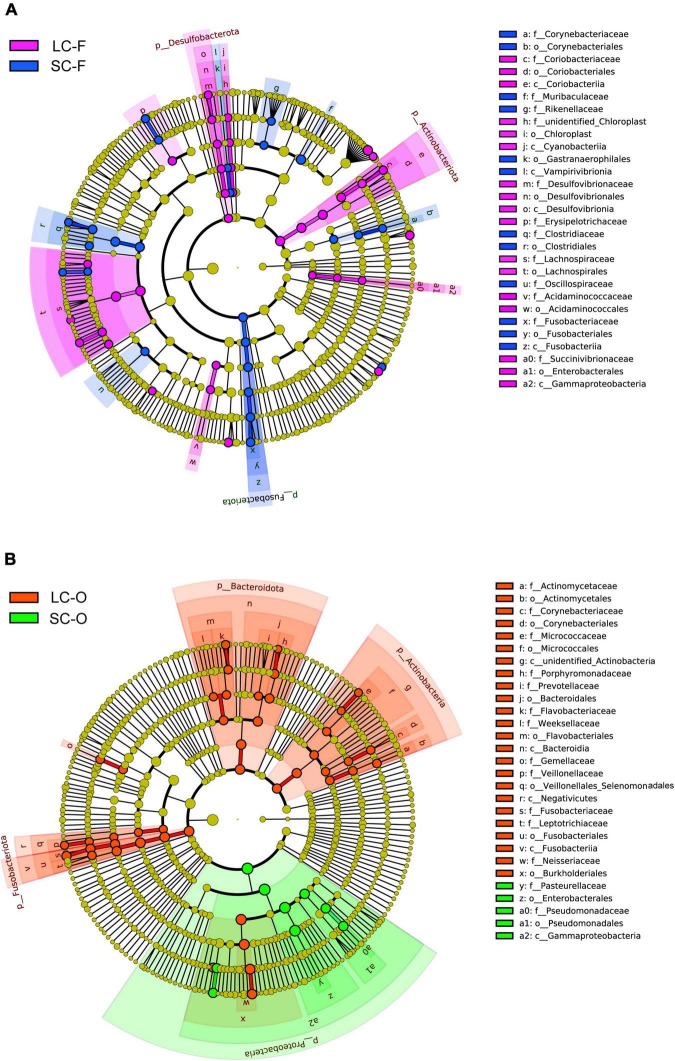
Linear discriminant analysis (LDA) effect size (LefSe) analysis. The cladogram shows the significantly differential taxa (LDA Score > 3, *P* < 0.05) in fecal **(A)** and oral microbiome **(B)** between the groups under long-term (LC) and short-term captivity (SC). Taxonomic ranks at the level of genus (g), family (f), order (o), class (c), and phylum (p) were arranged from the inside to the outside.

Linear discriminant analysis effect size analysis found 60 known bacterial taxa, explaining the differences in fecal microbiota between the groups (LDA > 3, *P* < 0.05; Supplementary Table 3, Figure 5A, and Supplementary Figure 2). The LC slow loris had significantly more Actinobacteriota and Desulfobacterota than the SC group, while Fusobacteriota was significantly enriched in the SC group. The LC group also had a high abundance of bacteria in Class Gammaproteobacteria and Orders Lachnospirales and Acidaminococcales. In contrast, Class Vampirivivrionia, Orders Corynebacteriales, Gastranaerophilales and Clostridiales, and Families Muribaculaceae, Rikenellaceae, and Oscillospiraceae were more abundant in the SC group. A total of 53 taxa were significantly enriched in the oral microbiota of a specific group, and the LC group had a larger number of statistically abundant taxa (*N* = 44) than the SC group (*N* = 9) (Supplementary Table 4, Figure 5B, and Supplementary Figure 2). We identified more abundant Actinobacteria, Bacteroidota, and Fusobacteriota in LC group, while a significantly greater abundance of Proteobacteria was observed in the SC group.

### Functional inference and significant difference analysis

**FIGURE 6:**
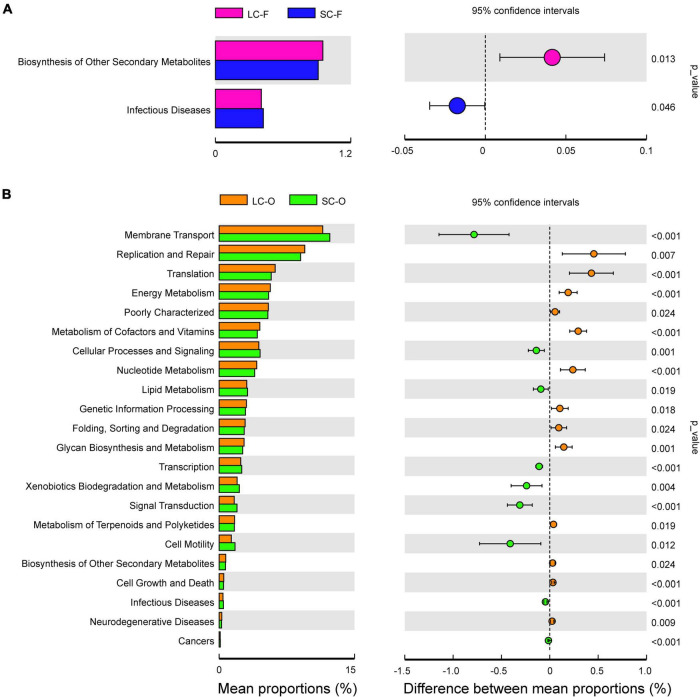
Phylogenetic Investigation of Communities by Reconstruction of Unobserved States (PICRUSt) analysis of the Kyoto Encyclopedia of Genes and Genomes (KEGG) metabolic pathways in the second level showed the significant differential functions of fecal **(A)** and oral microbiome **(B)** between long-term captivity (LC) and short-term captivity (SC) groups (tested by multiple *t*-test).

The variance analysis of Kyoto Encyclopedia of Genes and Genomes (KEGG) metabolic pathways was done to determine the functional gene composition *via* PICRUSt software. Only a few categories (level 2) displayed significant differences (*P* < 0.05) in fecal samples between groups (Figure 6A). A larger proportion of genes related to “Biosynthesis of other secondary metabolites” were found in the LC group (Supplementary Figure 3), while the genes related to “Infectious diseases” were significantly enriched in the SC group. In contrast, more significant differences in KEGG pathway abundance in the oral microbiome were detected (Figure 6B). The LC group was enriched with pathways in “Replication and repair,” “Translation,” and “Energy, Vitamins, and Nucleotide metabolism.” There were higher abundances of genes related to “Membrane transport,” “Cellular processes,” and “Xenobiotics biodegradation and metabolism” in the SC group, and a slightly higher abundance of “Lipid metabolism” and “Infectious diseases” was observed.

## Discussion

All the confiscated Bengal slow lorises were presumably wild-caught in this study. The captive individuals experienced many changes from the living environment in the wild to the human-constructed confinements. Both the gut and oral microbiome of confiscated Bengal slow lorises in the present study displayed significant alterations while considering the duration of captivity. These alterations were similar to the microbial shifts between wild and captive states reported in other studies. The slow loris under long-term captivities exhibited higher relative abundances of potential pathogens, such as *Desulfovibrio*, like the captive individuals in colobines (Amato et al., 2016) and sifakas (McKenney et al., 2017), implying an increased risk of gut microbial dysbiosis in serious captivity. In contrast, the butyrate or butyric acid-producing *Coprococcus eutactus* and *Clostridium butyricum* were highly enriched in short-term captive slow lorises. *Coprococcus* bacteria were associated with a higher quality of life indicators, depleted depression, and other cognitive outcomes (Kort et al., 2021). *C. butyricum*, which is generally found in the soil and intestines of healthy animals or humans, was reported as a potential probiotic balancing the intestinal microflora and stimulating the immune system (Zhang et al., 2016). Compared to the SC group, the LC group was significantly enriched by the genus *Collinsella*, which was associated with insulin resistance, obesity, and atherosclerosis in humans (Astbury et al., 2020; Houtz et al., 2021).

The gut microbiota between the LC and SC groups also showed different patterns from the captive-wild alterations reported in other species. Lachnospiraceae and Ruminococcaceae families, which are significantly abundant in wild monkeys, such as gray snub-nosed monkey (*Rhinopithecus brelichi*) (Hale et al., 2019), increased in long-term captive samples. These taxa were reported as prominent microbial community members in folivorous monkeys and were also associated with a plant fiber diet (Clayton et al., 2016). In addition to the gut bacteria, the high chloroplast content was reported in wild primates but decreased dramatically in captive populations (Clayton et al., 2018a). It was abundant in the slow lorises under long-term confinement. The results indicated that the confiscated animals had stable fiber intake under the current captive diet. It was consistent with the previous study by Ni et al. (2021), which indicated that apple peel and core might significantly contribute to the fiber content for the captive Bengal slow loris.

The slow lorises use plant exudate, such as tree gum, as a staple food resource in the wild (Cabana et al., 2017). *Bifidobacterium* has been reported as one of the most represented taxa in wild exudativorous primates (Brown et al., 2019; Cabana et al., 2019; Malukiewicz et al., 2022) as well as the captive lorises compared to some other nocturnal strepsirrhines (Bornbusch et al., 2019). As a potential biomarker of adaptation to exudivory, the Bifidobacteria group metabolizes arabinogalactan and pectin, the main components of carbohydrates in tree gum (Lugli et al., 2020). We observed that both LC and SC groups had a low abundance of *Bifidobacterium*, which is due to the scarcity of plant gum in their diet. Ni et al. (2021) reported that the increase of *Bifidobacterium* abundance in captive slow loris gut microbiota was positively correlated with the dietary intervention in supplying peach gum.

Compared to the fecal microbiome, the oral samples displayed higher Chao1 richness but lower Shannon and Simpson indices in this study, indicating that the oral microbial diversity was characterized by high taxa richness but less uniformity among samples based on Kers and Saccenti (2021). Consistently, the oral and fecal samples were clustered by the group based on different beta diversity metrics, and the oral microbiome exhibited higher community distance than the fecal microbiome. The high species richness and variation in the oral microbiome are related to the microbial acquisition of the oral cavity from the environment (Sawaswong et al., 2021).

The oral samples were highly characterized by pathogenic microbiota compared with the fecal samples, especially in the LC group. These pathogens are mostly associated with oral diseases. For example, the genera *Actinomyces*, *Capnocytophaga*, *Neisseria*, and *Fusobacterium*, and the species *Corynebacterium matruchotii* and *Porphyromonas gingivalis*, have been widely reported as causative agents of periodontitis, endodontic infections, and ulcerative gingivitis, which may destroy the periodontal tissues and lead to tooth loss (Hawkey et al., 1984; LeCorn et al., 2007; Nibali et al., 2020; Sedghi et al., 2021). *Rothia* spp. and *Capnocytophaga ochracea* have been identified as opportunistic pathogens causing septic arthritis, respiratory infection, and endocarditis (Jolivet-Gougeon et al., 2007; Tsuzukibashi et al., 2017). Some *Fusobacterium* and *Neisseria* species may also lead to adverse pregnancy outcomes (Sedghi et al., 2021). Because of the larger proportion of individuals with canine teeth loss and disability in the LC group, the captive duration may have detrimental effects on the oral and body health of Bengal slow lorises. However, there is no direct evidence of it in oral microbiota. Thus, further studies involving the diagnosis of pathogenic infections are highly recommended to identify the relationships between the oral microbiome and dental diseases.

The slow loris saliva can simulate protein sensitivity and cause an allergic reaction from physical contact like venom (Gardiner et al., 2018). The studies of the venomous host-associated microbiome, i.e., venom microbiomics, are burgeoning in addressing questions of how microorganisms colonize and evolve in venom glands (Ul-Hasan et al., 2019). For instance, the oral microbiota of venomous snakes may harbor potentially pathogenic groups that may cause post-bite infection (Smith et al., 2021). We found some distinct microbial communities in the SC group, e.g., *Pasteurella*, which are the most common taxa isolated from infected bite wounds of many animal species (Abrahamian and Goldstein, 2011), indicating that there are some bite-related pathogenic agents in slow lorises’ oral flora.

The inference of functional analysis revealed that the OTUs related to metabolism pathways (e.g., cofactors, vitamins, and nucleotide metabolism) were significantly abundant in the fecal and oral microbiome of the LC group, which implies that the long-term captivity may be more efficient in ensuring the host to digest and absorb in a stable state (Ning et al., 2020). The pathways of “membrane transport” and “cell motility,” enriched in oral microbiota of the SC group, are more abundant in free-ranging animals than in captive individuals due to various food supplies and environments (Menke et al., 2017). In addition, the relative abundance of genes related to xenobiotic metabolism and degradation pathway in the SC group may imply that the individuals under short-term confinement still retain some native microbiota related to a gummivorous diet, as gums are loaded with plant secondary metabolites and the related microbes contain detoxification pathways (Cabana et al., 2019).

## Conclusion

In this study, we characterized the oral microbiome of Lorisidae primate species and analyzed the fecal-oral microbial variation between two groups under different confinement durations to determine the effects of captivity on Bengal slow lorises. The gut and oral microbial variations showed similar patterns to the wild-captive differences reported in other species. The results indicated that the confiscated Bengal slow lorises under current husbandry management, particularly those with the extended confinements, were unsuitable for reintroducing into the wild. Compared to the gut microbiome, the oral microbes were more sensitive in response to the captive duration. Our study suggests that the oral microbiome can be used as a regular tool in monitoring the host status during the reintroduction process.

## Data availability statement

The data presented in this study are deposited in the National Genomics Data Center (NGDC) (https://ngdc.cncb.ac.cn/), accession numbers: PRJCA009497 and PRJCA009498.

## Ethics statement

This animal study was reviewed and approved by the Institutional Review Board (IRB13627) and the Institutional Animal Care and Use Committee of the Sichuan Agricultural University, China.

## Author contributions

QN conceived and designed the experiments, performed the experiments, analyzed the data, prepared figures and/or table, and authored or reviewed drafts of the manuscript. SD, BX, and MX performed the experiments, analyzed the data, and prepared figures and/or table. BZ, FK, HX, DL, YY, MZ, XF, DY, and MY analyzed the data and contributed reagents, materials, and analysis tools. All authors contributed to the article and approved the submitted version.

## Footnotes

1
http://ccb.jhu.edu/software/FLASH/

2
http://qiime.org/scripts/split_libraries_fastq.html

3
https://www.arb-silva.de/

4
http://www.drive5.com/usearch/manual/uchime_algo.html

5
https://ngdc.cncb.ac.cn/

6
http://drive5.com/uparse/
