# Supplementary material for: Oral and fecal microbiome of confiscated Bengal slow lorises in response to confinement duration
Source: Front Microbiol. 2022 Sep 27;13:941261. doi: 10.3389/fmicb.2022.941261 (PMC9553000; doi:10.3389/fmicb.2022.941261)
Supplement: Supplementary file 1 [file Data_Sheet_1.docx]

Supplementary Material

# Supplementary Figures and Tables

## Supplementary Tables

Supplementary Table 1 OTU summary of the fecal and oral samples. *LC-F* (long-term captivity-fecal), *SC-F* (short-term captivity-fecal), *LC-O* (long-term captivity-oral), *SC-O* (short-term captivity-oral).

| Sample Name | Raw PE(#) | Raw Tags(#) | Clean Tags(#) | Effective Tags(#) | Base(nt) | AvgLen(nt) | Q20 | Q30 | GC% | Effective% |
| --- | --- | --- | --- | --- | --- | --- | --- | --- | --- | --- |
| LC-F01 | 82,892 | 82,319 | 82,028 | 71,185 | 18,001,592 | 253 | 99.38 | 97.98 | 50.58 | 85.88 |
| LC-F02 | 87,680 | 87,614 | 87,434 | 80,259 | 20,283,592 | 253 | 99.38 | 97.94 | 57.22 | 91.54 |
| LC-F03 | 82,879 | 81,262 | 80,880 | 68,619 | 17,338,156 | 253 | 99.53 | 98.44 | 56.61 | 82.79 |
| LC-F04 | 90,546 | 89,460 | 89,018 | 75,364 | 19,049,686 | 253 | 99.33 | 97.85 | 55.5 | 83.23 |
| LC-F05 | 91,073 | 90,045 | 89,608 | 83,392 | 21,092,924 | 253 | 99.43 | 98.14 | 53.67 | 91.57 |
| LC-F06 | 92,345 | 91,713 | 91,224 | 76,883 | 19,439,071 | 253 | 99.35 | 97.89 | 52.55 | 83.26 |
| LC-F07 | 88,942 | 87,637 | 86,890 | 70,266 | 17,761,858 | 253 | 99.38 | 97.98 | 53.1 | 79 |
| LC-F08 | 89,753 | 88,134 | 87,548 | 73,371 | 18,546,548 | 253 | 99.41 | 98.11 | 54.97 | 81.75 |
| LC-F09 | 77,855 | 75,983 | 75,352 | 63,440 | 16,036,483 | 253 | 99.36 | 97.99 | 55.46 | 81.48 |
| LC-F10 | 90,603 | 89,580 | 89,068 | 73,171 | 18,482,181 | 253 | 99.39 | 98.04 | 56.49 | 80.76 |
| LC-F11 | 86,671 | 86,293 | 86,010 | 73,909 | 18,676,842 | 253 | 99.42 | 98.07 | 56.33 | 85.28 |
| LC-F12 | 86,420 | 86,252 | 85,979 | 75,177 | 18,987,712 | 253 | 99.37 | 97.94 | 54.28 | 86.99 |
| LC-F13 | 79,678 | 78,450 | 78,054 | 63,208 | 15,982,441 | 253 | 99.43 | 98.13 | 52.97 | 79.33 |
| LC-F14 | 87,821 | 87,241 | 86,926 | 71,437 | 18,058,392 | 253 | 99.43 | 98.14 | 53.15 | 81.34 |
| LC-F15 | 81,925 | 80,455 | 80,063 | 65,368 | 16,516,129 | 253 | 99.43 | 98.14 | 52.09 | 79.79 |
| LC-F16 | 86,791 | 85,656 | 85,298 | 69,569 | 17,577,288 | 253 | 99.43 | 98.15 | 56 | 80.16 |
| LC-F17 | 94,314 | 93,648 | 93,319 | 78,890 | 19,936,152 | 253 | 99.44 | 98.15 | 55.77 | 83.65 |
| LC-F18 | 89,747 | 89,569 | 89,239 | 75,751 | 19,146,389 | 253 | 99.41 | 98.07 | 55.36 | 84.41 |
| LC-F19 | 82,426 | 82,368 | 82,152 | 70,101 | 17,724,797 | 253 | 99.48 | 98.23 | 54.05 | 85.05 |
| LC-F20 | 80,211 | 79,889 | 79,617 | 65,417 | 16,536,904 | 253 | 99.4 | 98.06 | 52.8 | 81.56 |
| LC-F21 | 89,857 | 88,925 | 88,457 | 75,988 | 19,210,158 | 253 | 99.38 | 98.01 | 55.41 | 84.57 |
| LC-F22 | 87,150 | 85,735 | 85,123 | 72,623 | 18,353,771 | 253 | 99.35 | 97.92 | 55.92 | 83.33 |
| LC-F23 | 91,486 | 91,364 | 91,100 | 80,145 | 20,262,173 | 253 | 99.46 | 98.15 | 53.64 | 87.6 |
| LC-F24 | 92,863 | 91,854 | 91,359 | 77,153 | 19,500,490 | 253 | 99.31 | 97.82 | 53.96 | 83.08 |
| LC-F25 | 86,108 | 85,367 | 85,035 | 70,941 | 17,936,846 | 253 | 99.42 | 98.1 | 52.79 | 82.39 |
| LC-F26 | 83,186 | 82,411 | 82,014 | 69,121 | 17,464,740 | 253 | 99.37 | 97.98 | 53.68 | 83.09 |
| LC-F27 | 91,009 | 90,456 | 90,138 | 72,524 | 18,326,207 | 253 | 99.4 | 98.03 | 51.72 | 79.69 |
| LC-F28 | 81,285 | 79,920 | 79,491 | 61,883 | 15,631,044 | 253 | 99.36 | 97.99 | 52.81 | 76.13 |
| LC-F29 | 77,489 | 77,241 | 76,974 | 68,890 | 17,401,238 | 253 | 99.32 | 97.82 | 57.6 | 88.9 |
| LC-F30 | 91,073 | 89,676 | 89,306 | 77,458 | 19,590,378 | 253 | 99.55 | 98.54 | 54.04 | 85.05 |
| LC-F31 | 84,424 | 83,236 | 82,860 | 75,018 | 18,968,051 | 253 | 99.53 | 98.46 | 56.18 | 88.86 |
| LC-F32 | 89,876 | 88,755 | 88,460 | 78,275 | 19,795,691 | 253 | 99.54 | 98.53 | 52.09 | 87.09 |
| LC-F33 | 84,926 | 84,550 | 84,350 | 75,746 | 19,149,715 | 253 | 99.51 | 98.42 | 55.36 | 89.19 |
| LC-F34 | 83,638 | 82,670 | 82,375 | 74,525 | 18,831,673 | 253 | 99.47 | 98.29 | 53.18 | 89.1 |
| SC-F35 | 91,314 | 90,530 | 90,303 | 78,416 | 19,806,558 | 253 | 99.5 | 98.43 | 52.25 | 85.88 |
| SC-F36 | 83,427 | 83,340 | 83,216 | 74,147 | 18,741,677 | 253 | 99.59 | 98.6 | 52.87 | 88.88 |
| SC-F37 | 80,889 | 78,992 | 78,615 | 68,166 | 17,235,600 | 253 | 99.56 | 98.54 | 52.49 | 84.27 |
| SC-F38 | 89,147 | 88,526 | 88,280 | 78,794 | 19,919,383 | 253 | 99.51 | 98.43 | 53.5 | 88.39 |
| SC-F39 | 88,936 | 88,225 | 87,989 | 80,107 | 20,245,316 | 253 | 99.46 | 98.17 | 55.75 | 90.07 |
| SC-F40 | 95,141 | 94,178 | 93,747 | 85,917 | 21,713,958 | 253 | 99.4 | 98.07 | 55.24 | 90.3 |
| SC-F41 | 84,018 | 83,007 | 82,608 | 73,145 | 18,492,953 | 253 | 99.42 | 98.12 | 55.63 | 87.06 |
| SC-F42 | 87,226 | 86,425 | 86,113 | 75,410 | 19,066,212 | 253 | 99.45 | 98.16 | 54.42 | 86.45 |
| SC-F43 | 87,367 | 86,316 | 86,021 | 76,050 | 19,225,455 | 253 | 99.46 | 98.18 | 54.47 | 87.05 |
| SC-F44 | 80,574 | 79,735 | 79,414 | 67,486 | 17,050,585 | 253 | 99.43 | 98.09 | 56.1 | 83.76 |
| SC-F45 | 82,441 | 81,410 | 80,992 | 69,298 | 17,523,944 | 253 | 99.35 | 97.91 | 53.26 | 84.06 |
| SC-F46 | 84,514 | 83,981 | 83,770 | 73,502 | 18,585,029 | 253 | 99.47 | 98.19 | 53.8 | 86.97 |
| SC-F47 | 87,657 | 86,685 | 86,357 | 78,716 | 19,904,037 | 253 | 99.47 | 98.21 | 55.25 | 89.8 |
| SC-F48 | 91,475 | 90,947 | 90,779 | 80,627 | 20,373,965 | 253 | 99.47 | 98.17 | 55.2 | 88.14 |
| SC-F49 | 87,373 | 85,746 | 85,413 | 75,713 | 19,138,874 | 253 | 99.45 | 98.17 | 55.09 | 86.65 |
| SC-F50 | 89,370 | 89,213 | 88,929 | 82,316 | 20,805,607 | 253 | 99.47 | 98.2 | 54.45 | 92.11 |
| SC-F51 | 80,350 | 79,799 | 79,263 | 73,880 | 18,688,142 | 253 | 99.41 | 98.12 | 54.23 | 91.95 |
| SC-F52 | 91,963 | 91,536 | 91,367 | 77,541 | 19,604,251 | 253 | 99.47 | 98.18 | 55.41 | 84.32 |
| SC-F53 | 86,715 | 86,401 | 86,164 | 75,215 | 19,010,298 | 253 | 99.4 | 98 | 53.66 | 86.74 |
| SC-F54 | 90,773 | 90,614 | 90,462 | 78,424 | 19,811,479 | 253 | 99.38 | 97.93 | 52.25 | 86.4 |
| SC-F55 | 85,532 | 83,988 | 83,522 | 73,837 | 18,659,985 | 253 | 99.34 | 97.86 | 56.04 | 86.33 |
| SC-F56 | 97,097 | 96,265 | 95,929 | 81,694 | 20,657,965 | 253 | 99.39 | 97.99 | 53.24 | 84.14 |
| SC-F57 | 86,605 | 85,938 | 85,570 | 68,944 | 17,447,787 | 253 | 99.27 | 97.64 | 54.54 | 79.61 |
| SC-F58 | 92,168 | 91,233 | 90,843 | 78,626 | 19,875,584 | 253 | 99.42 | 98.07 | 56.39 | 85.31 |
| SC-F59 | 95,776 | 95,183 | 94,884 | 85,757 | 21,679,936 | 253 | 99.39 | 97.98 | 53.46 | 89.54 |
| SC-F60 | 88,907 | 88,545 | 88,368 | 77,516 | 19,588,338 | 253 | 99.42 | 98.05 | 53.93 | 87.19 |
| SC-F61 | 87,940 | 87,048 | 86,678 | 77,084 | 19,486,976 | 253 | 99.35 | 97.87 | 56.77 | 87.66 |
| SC-F62 | 81,635 | 80,761 | 80,405 | 70,051 | 17,708,769 | 253 | 99.36 | 97.9 | 54.42 | 85.81 |
| SC-F63 | 90,477 | 89,446 | 88,939 | 79,663 | 20,131,524 | 253 | 99.3 | 97.75 | 56.26 | 88.05 |
| SC-F64 | 90,053 | 88,816 | 88,360 | 79,091 | 19,997,399 | 253 | 99.38 | 97.95 | 57.18 | 87.83 |
| SC-F65 | 86,921 | 85,797 | 85,303 | 71,900 | 18,179,960 | 253 | 99.4 | 98.04 | 53.96 | 82.72 |
| SC-F66 | 80,737 | 80,654 | 80,532 | 69,089 | 17,467,892 | 253 | 99.49 | 98.23 | 53.34 | 85.57 |
| SC-F67 | 96,185 | 95,580 | 95,242 | 84,825 | 21,422,603 | 253 | 99.43 | 98.1 | 54.51 | 88.19 |
| SC-F68 | 81,673 | 81,625 | 81,499 | 64,510 | 16,304,986 | 253 | 99.33 | 97.81 | 53.87 | 78.99 |
| SC-F69 | 82,048 | 80,728 | 80,388 | 72,095 | 18,229,853 | 253 | 99.47 | 98.22 | 54.29 | 87.87 |
| SC-F70 | 82,049 | 81,901 | 81,712 | 75,493 | 19,081,865 | 253 | 99.44 | 98.14 | 54.21 | 92.01 |
| SC-F71 | 77,233 | 76,334 | 75,923 | 72,184 | 18,249,077 | 253 | 99.36 | 97.9 | 58.13 | 93.46 |
| SC-F72 | 82,817 | 82,229 | 82,013 | 71,825 | 18,153,427 | 253 | 99.42 | 98.06 | 54.18 | 86.73 |
| SC-F73 | 79,375 | 78,337 | 78,004 | 67,128 | 16,958,684 | 253 | 99.42 | 98.08 | 54.81 | 84.57 |
| SC-F74 | 88,100 | 87,643 | 87,438 | 77,070 | 19,476,685 | 253 | 99.4 | 98.01 | 55.28 | 87.48 |
| SC-F75 | 82,971 | 81,514 | 81,041 | 70,069 | 17,719,106 | 253 | 99.42 | 98.09 | 55.1 | 84.45 |
| SC-F76 | 95,329 | 94,692 | 94,278 | 77,640 | 19,631,743 | 253 | 99.44 | 98.14 | 53.38 | 81.44 |
| SC-F77 | 82,248 | 81,681 | 81,368 | 67,913 | 17,175,281 | 253 | 99.41 | 98.08 | 51.39 | 82.57 |
| LC-O01 | 80,381 | 80,230 | 80,110 | 67,799 | 17,149,519 | 253 | 99.59 | 98.61 | 51.53 | 84.35 |
| LC-O02 | 80,971 | 80,650 | 80,491 | 67,840 | 17,162,126 | 253 | 99.53 | 98.43 | 52.46 | 83.78 |
| LC-O03 | 87,410 | 87,350 | 87,241 | 74,877 | 18,942,332 | 253 | 99.6 | 98.63 | 52.13 | 85.66 |
| LC-O04 | 87,920 | 87,390 | 87,068 | 72,402 | 18,311,209 | 253 | 99.59 | 98.62 | 52.14 | 82.35 |
| LC-O05 | 86,280 | 85,837 | 85,511 | 73,380 | 18,560,950 | 253 | 99.52 | 98.43 | 52.46 | 85.05 |
| LC-O06 | 84,703 | 84,363 | 84,054 | 71,423 | 18,068,556 | 253 | 99.61 | 98.67 | 52.65 | 84.32 |
| LC-O07 | 81,236 | 79,798 | 79,476 | 67,823 | 17,149,583 | 253 | 99.58 | 98.61 | 52.61 | 83.49 |
| LC-O08 | 79,994 | 79,519 | 79,184 | 68,995 | 17,454,051 | 253 | 99.54 | 98.46 | 50.86 | 86.25 |
| LC-O09 | 75,879 | 75,715 | 75,598 | 63,522 | 16,064,235 | 253 | 99.6 | 98.63 | 51.85 | 83.71 |
| LC-O10 | 89,534 | 89,001 | 88,685 | 75,476 | 19,094,003 | 253 | 99.6 | 98.66 | 52.06 | 84.3 |
| LC-O11 | 89,561 | 88,929 | 88,708 | 75,998 | 19,261,418 | 253 | 99.56 | 98.57 | 52 | 84.86 |
| LC-O12 | 89,277 | 88,899 | 88,735 | 78,171 | 19,837,047 | 254 | 99.64 | 98.75 | 52.8 | 87.56 |
| LC-O13 | 86,674 | 86,341 | 86,143 | 67,512 | 17,097,172 | 253 | 99.65 | 98.77 | 53.84 | 77.89 |
| LC-O14 | 92,465 | 90,797 | 90,599 | 78,684 | 19,923,212 | 253 | 99.57 | 98.6 | 52.21 | 85.1 |
| LC-O15 | 94,738 | 93,506 | 93,162 | 79,550 | 20,158,354 | 253 | 99.55 | 98.49 | 52.65 | 83.97 |
| LC-O16 | 82,448 | 81,372 | 81,193 | 71,341 | 18,081,274 | 253 | 99.6 | 98.7 | 52.25 | 86.53 |
| LC-O17 | 85,738 | 85,236 | 85,109 | 75,438 | 19,100,514 | 253 | 99.64 | 98.74 | 52.11 | 87.99 |
| LC-O18 | 84,822 | 84,651 | 84,527 | 74,671 | 18,938,405 | 254 | 99.66 | 98.79 | 52.58 | 88.03 |
| LC-O19 | 84,541 | 83,269 | 83,021 | 71,504 | 18,158,643 | 254 | 99.58 | 98.59 | 52.9 | 84.58 |
| LC-O20 | 83,667 | 82,319 | 82,100 | 72,777 | 18,455,456 | 254 | 99.61 | 98.67 | 53.09 | 86.98 |
| LC-O21 | 80,808 | 80,095 | 79,802 | 65,291 | 16,537,618 | 253 | 99.6 | 98.68 | 53.12 | 80.8 |
| LC-O22 | 92,578 | 90,977 | 90,803 | 77,353 | 19,571,137 | 253 | 99.63 | 98.71 | 52.64 | 83.55 |
| LC-O23 | 86,853 | 84,339 | 84,009 | 75,388 | 19,153,030 | 254 | 99.47 | 98.31 | 52.68 | 86.8 |
| LC-O24 | 97,006 | 96,057 | 95,740 | 86,180 | 21,834,134 | 253 | 99.53 | 98.45 | 52.73 | 88.84 |
| LC-O25 | 76,904 | 76,207 | 75,976 | 61,363 | 15,595,785 | 254 | 99.55 | 98.47 | 53.96 | 79.79 |
| LC-O26 | 88,040 | 87,017 | 86,734 | 75,251 | 19,042,115 | 253 | 99.55 | 98.54 | 52.63 | 85.47 |
| LC-O27 | 80,377 | 78,950 | 78,536 | 68,514 | 17,370,530 | 254 | 99.44 | 98.19 | 52.91 | 85.24 |
| LC-O28 | 87,445 | 85,850 | 85,556 | 74,971 | 18,964,650 | 253 | 99.58 | 98.6 | 52.45 | 85.74 |
| LC-O29 | 91,045 | 89,785 | 89,458 | 77,577 | 19,632,263 | 253 | 99.48 | 98.38 | 51.93 | 85.21 |
| LC-O30 | 87,541 | 85,321 | 84,930 | 75,186 | 19,080,413 | 254 | 99.54 | 98.46 | 52.52 | 85.89 |
| LC-O31 | 83,235 | 80,926 | 80,422 | 69,567 | 17,692,056 | 254 | 99.48 | 98.31 | 52.66 | 83.58 |
| LC-O32 | 84,037 | 83,592 | 83,354 | 73,953 | 18,738,566 | 253 | 99.55 | 98.47 | 52.95 | 88 |
| LC-O33 | 90,153 | 88,887 | 88,436 | 78,438 | 19,863,010 | 253 | 99.54 | 98.47 | 52.19 | 87.01 |
| LC-O34 | 88,892 | 87,150 | 86,889 | 75,869 | 19,274,854 | 254 | 99.53 | 98.43 | 52.41 | 85.35 |
| LC-O35 | 88,434 | 85,755 | 85,464 | 77,106 | 19,543,953 | 253 | 99.4 | 98.08 | 52.7 | 87.19 |
| LC-O36 | 87,914 | 87,740 | 87,545 | 79,843 | 20,331,821 | 255 | 99.52 | 98.41 | 53.12 | 90.82 |
| LC-O37 | 86,502 | 85,023 | 84,708 | 70,750 | 17,926,320 | 253 | 99.61 | 98.66 | 53.34 | 81.79 |
| SC-O38 | 89,465 | 87,053 | 86,852 | 80,103 | 20,272,520 | 253 | 99.61 | 98.72 | 52.51 | 89.54 |
| SC-O39 | 93,034 | 91,523 | 91,228 | 83,752 | 21,241,397 | 254 | 99.47 | 98.32 | 52.27 | 90.02 |
| SC-O40 | 72,248 | 61,838 | 61,189 | 52,682 | 12,944,018 | 246 | 99.38 | 98.03 | 52.62 | 72.92 |
| SC-O41 | 87,818 | 81,642 | 81,446 | 74,162 | 18,756,090 | 253 | 99.56 | 98.57 | 52.26 | 84.45 |
| SC-O42 | 90,583 | 88,575 | 88,338 | 80,592 | 20,432,850 | 254 | 99.56 | 98.58 | 52.37 | 88.97 |
| SC-O43 | 83,557 | 82,888 | 82,686 | 74,070 | 18,745,131 | 253 | 99.56 | 98.54 | 52.45 | 88.65 |
| SC-O44 | 94,513 | 94,246 | 94,118 | 84,395 | 21,368,444 | 253 | 99.6 | 98.68 | 52.94 | 89.29 |
| SC-O45 | 87,441 | 86,828 | 86,617 | 70,830 | 17,904,536 | 253 | 99.58 | 98.61 | 52.31 | 81 |
| SC-O46 | 83,076 | 72,749 | 72,139 | 61,428 | 15,136,551 | 246 | 99.41 | 98.1 | 52.75 | 73.94 |
| SC-O47 | 85,395 | 84,479 | 84,236 | 76,103 | 19,298,615 | 254 | 99.52 | 98.48 | 52.08 | 89.12 |
| SC-O48 | 86,539 | 86,468 | 86,302 | 76,260 | 19,368,823 | 254 | 99.58 | 98.6 | 52.64 | 88.12 |
| SC-O49 | 84,067 | 83,823 | 83,663 | 66,828 | 17,024,242 | 255 | 99.64 | 98.74 | 55.05 | 79.49 |
| SC-O50 | 79,965 | 79,693 | 79,564 | 71,483 | 18,106,324 | 253 | 99.61 | 98.71 | 52.32 | 89.39 |
| SC-O51 | 87,239 | 86,490 | 86,267 | 75,550 | 19,163,483 | 254 | 99.57 | 98.53 | 52.38 | 86.6 |
| SC-O52 | 85,157 | 84,150 | 83,960 | 74,886 | 18,962,843 | 253 | 99.59 | 98.67 | 52.12 | 87.94 |
| SC-O53 | 89,984 | 88,365 | 88,113 | 79,681 | 20,157,428 | 253 | 99.61 | 98.74 | 52.12 | 88.55 |
| SC-O54 | 91,700 | 91,073 | 90,750 | 69,614 | 17,610,829 | 253 | 99.63 | 98.74 | 52.16 | 75.91 |
| SC-O55 | 86,814 | 85,979 | 85,697 | 74,920 | 18,983,000 | 253 | 99.58 | 98.62 | 52.56 | 86.3 |
| SC-O56 | 87,768 | 86,798 | 86,509 | 78,566 | 19,887,090 | 253 | 99.65 | 98.81 | 52.4 | 89.52 |
| SC-O57 | 84,163 | 83,894 | 83,684 | 73,724 | 18,637,549 | 253 | 99.6 | 98.66 | 52.39 | 87.6 |
| SC-O58 | 85,601 | 84,223 | 83,986 | 71,701 | 18,132,987 | 253 | 99.58 | 98.63 | 52.12 | 83.76 |
| SC-O59 | 80,186 | 79,408 | 79,212 | 69,550 | 17,657,630 | 254 | 99.56 | 98.54 | 52.63 | 86.74 |
| SC-O60 | 85,133 | 83,826 | 83,427 | 72,154 | 18,297,369 | 254 | 99.48 | 98.39 | 52.45 | 84.75 |
| SC-O61 | 92,306 | 92,293 | 92,155 | 78,603 | 19,938,213 | 254 | 99.61 | 98.67 | 53.41 | 85.15 |
| SC-O62 | 83,023 | 81,735 | 81,493 | 72,393 | 18,339,905 | 253 | 99.63 | 98.76 | 52.35 | 87.2 |
| SC-O63 | 92,134 | 91,504 | 91,309 | 79,679 | 20,246,270 | 254 | 99.53 | 98.43 | 52.86 | 86.48 |
| SC-O64 | 88,791 | 87,819 | 87,495 | 74,941 | 19,025,827 | 254 | 99.6 | 98.69 | 52.42 | 84.4 |
| SC-O65 | 85,955 | 85,550 | 85,328 | 75,240 | 19,087,461 | 254 | 99.53 | 98.49 | 52.26 | 87.53 |
| SC-O66 | 80,964 | 80,130 | 79,963 | 69,886 | 17,755,907 | 254 | 99.62 | 98.69 | 52.66 | 86.32 |
| SC-O67 | 88,199 | 86,310 | 86,020 | 76,080 | 19,315,009 | 254 | 99.65 | 98.78 | 52.56 | 86.26 |
| SC-O68 | 91,727 | 91,383 | 91,132 | 75,915 | 19,208,218 | 253 | 99.63 | 98.75 | 52.5 | 82.76 |
| SC-O69 | 90,864 | 89,279 | 88,848 | 77,474 | 19,652,267 | 254 | 99.6 | 98.67 | 52.97 | 85.26 |
| SC-O70 | 87,523 | 86,702 | 86,415 | 70,504 | 17,831,379 | 253 | 99.63 | 98.73 | 52.45 | 80.55 |
| SC-O71 | 90,550 | 90,107 | 89,924 | 78,488 | 19,950,125 | 254 | 99.62 | 98.7 | 52.38 | 86.68 |
| SC-O72 | 85,942 | 85,515 | 85,308 | 72,662 | 18,390,120 | 253 | 99.62 | 98.72 | 52.48 | 84.55 |
| SC-O73 | 90,328 | 89,617 | 89,374 | 73,046 | 18,488,228 | 253 | 99.62 | 98.73 | 52.74 | 80.87 |
| SC-O74 | 85,627 | 84,516 | 84,355 | 76,287 | 19,320,964 | 253 | 99.43 | 98.26 | 51.54 | 89.09 |
| SC-O75 | 93,239 | 92,641 | 92,423 | 77,696 | 19,689,937 | 253 | 99.51 | 98.43 | 52.06 | 83.33 |
| SC-O76 | 85,619 | 84,896 | 84,631 | 71,302 | 18,053,247 | 253 | 99.65 | 98.82 | 52.18 | 83.28 |
| SC-O77 | 81,902 | 80,641 | 80,352 | 70,198 | 17,769,710 | 253 | 99.65 | 98.82 | 52.43 | 85.71 |
| SC-O78 | 83,900 | 83,311 | 83,077 | 65,927 | 16,689,252 | 253 | 99.64 | 98.78 | 53.09 | 78.58 |
| SC-O79 | 77,047 | 76,132 | 75,895 | 62,272 | 15,770,651 | 253 | 99.66 | 98.84 | 52.71 | 80.82 |
| SC-O80 | 82,063 | 81,485 | 81,226 | 65,450 | 16,568,191 | 253 | 99.57 | 98.62 | 52.84 | 79.76 |
| SC-O81 | 85,567 | 84,541 | 84,204 | 72,583 | 18,398,405 | 253 | 99.62 | 98.72 | 53.18 | 84.83 |
| SC-O82 | 94,370 | 91,550 | 91,351 | 79,314 | 20,073,736 | 253 | 99.64 | 98.75 | 53.12 | 84.05 |
| SC-O83 | 87,602 | 85,972 | 85,663 | 76,564 | 19,404,301 | 253 | 99.48 | 98.34 | 54.13 | 87.4 |
| SC-O84 | 93,477 | 93,418 | 93,279 | 80,835 | 20,499,826 | 254 | 99.48 | 98.41 | 53.28 | 86.48 |
| SC-O85 | 83,885 | 78,155 | 77,914 | 67,540 | 17,203,911 | 255 | 99.46 | 98.34 | 54.6 | 80.51 |

Supplementary Table 2 Summary of the Alpha diversity of the fecal and oral microbiome.

| Sample Name | observed_species | shannon | simpson | chao1 | ACE | goods_coverage | PD_whole_tree |
| --- | --- | --- | --- | --- | --- | --- | --- |
| LC-F1 | 664 | 5.853 | 0.936 | 722 | 746.814 | 0.998 | 52.456 |
| LC-F2 | 228 | 3.82 | 0.842 | 309.053 | 275.894 | 0.999 | 20.315 |
| LC-F3 | 543 | 4.849 | 0.894 | 579.695 | 596.534 | 0.998 | 49.286 |
| LC-F4 | 540 | 4.919 | 0.902 | 655.957 | 642.635 | 0.997 | 42.26 |
| LC-F5 | 391 | 3.107 | 0.751 | 474.081 | 485.353 | 0.998 | 39.989 |
| LC-F6 | 538 | 5.292 | 0.933 | 657.418 | 647.241 | 0.997 | 38.498 |
| LC-F7 | 587 | 5.612 | 0.93 | 646.221 | 651.451 | 0.998 | 48.205 |
| LC-F8 | 596 | 5.683 | 0.95 | 697.68 | 696.686 | 0.997 | 48.485 |
| LC-F9 | 469 | 5.129 | 0.92 | 518.8 | 533.357 | 0.998 | 39.635 |
| LC-F10 | 520 | 5.355 | 0.941 | 598.765 | 596.772 | 0.998 | 47.296 |
| LC-F11 | 508 | 5.292 | 0.942 | 580.958 | 585.94 | 0.998 | 43.22 |
| LC-F12 | 523 | 4.615 | 0.873 | 634.167 | 614.983 | 0.998 | 45.179 |
| LC-F13 | 694 | 6.332 | 0.951 | 768.489 | 778.997 | 0.998 | 54.309 |
| LC-F14 | 635 | 5.92 | 0.951 | 713.471 | 717.801 | 0.998 | 50.271 |
| LC-F15 | 613 | 5.948 | 0.955 | 668.883 | 688.874 | 0.998 | 45.589 |
| LC-F16 | 541 | 5.591 | 0.952 | 577.943 | 606.278 | 0.998 | 40.21 |
| LC-F17 | 490 | 5.213 | 0.93 | 560.545 | 560.028 | 0.998 | 41.991 |
| LC-F18 | 554 | 5.585 | 0.94 | 647.923 | 638.555 | 0.998 | 48.46 |
| LC-F19 | 608 | 5.179 | 0.905 | 653.778 | 688.768 | 0.998 | 49.793 |
| LC-F20 | 562 | 5.763 | 0.945 | 599.396 | 618.407 | 0.998 | 46.542 |
| LC-F21 | 455 | 5.062 | 0.924 | 526.842 | 523.663 | 0.998 | 40.345 |
| LC-F22 | 507 | 5.473 | 0.95 | 593.625 | 579.013 | 0.998 | 45.484 |
| LC-F23 | 513 | 4.65 | 0.874 | 606.613 | 617.495 | 0.997 | 39.986 |
| LC-F24 | 515 | 5.811 | 0.962 | 598.081 | 592.44 | 0.998 | 44.387 |
| LC-F25 | 621 | 6.005 | 0.958 | 685.75 | 707.786 | 0.998 | 51.742 |
| LC-F26 | 589 | 5.51 | 0.938 | 642.027 | 667.685 | 0.998 | 46.534 |
| LC-F27 | 540 | 5.399 | 0.933 | 616.233 | 627.759 | 0.998 | 45.833 |
| LC-F28 | 522 | 5.772 | 0.951 | 580.043 | 585.845 | 0.998 | 42.28 |
| LC-F29 | 395 | 4.671 | 0.896 | 425.09 | 435.061 | 0.999 | 37.389 |
| LC-F30 | 1068 | 5.565 | 0.939 | 1437.005 | 1464.633 | 0.992 | 94.94 |
| LC-F31 | 916 | 4.89 | 0.905 | 1225.094 | 1214.837 | 0.993 | 82.817 |
| LC-F32 | 560 | 4.203 | 0.799 | 684.937 | 693.172 | 0.997 | 57.187 |
| LC-F33 | 552 | 5.764 | 0.955 | 717.362 | 692.963 | 0.997 | 50.538 |
| LC-F34 | 652 | 6.191 | 0.962 | 787 | 760.022 | 0.997 | 57.626 |
| SC-F35 | 564 | 5.067 | 0.891 | 762.722 | 708.55 | 0.997 | 50.243 |
| SC-F36 | 647 | 5.723 | 0.936 | 802.296 | 770.463 | 0.997 | 55.793 |
| SC-F37 | 655 | 6.091 | 0.959 | 712 | 745.128 | 0.998 | 56.07 |
| SC-F38 | 556 | 4.92 | 0.899 | 671.28 | 667.188 | 0.997 | 47.308 |
| SC-F39 | 634 | 5.53 | 0.95 | 787.632 | 775.577 | 0.997 | 46.838 |
| SC-F40 | 564 | 5.464 | 0.949 | 660.041 | 688.339 | 0.997 | 46.461 |
| SC-F41 | 526 | 5.902 | 0.966 | 631 | 604.23 | 0.998 | 42.078 |
| SC-F42 | 608 | 5.773 | 0.952 | 692.733 | 706.075 | 0.997 | 49.722 |
| SC-F43 | 642 | 5.51 | 0.928 | 795.529 | 766.682 | 0.997 | 45.93 |
| SC-F44 | 567 | 5.815 | 0.957 | 620.723 | 641.42 | 0.998 | 46.905 |
| SC-F45 | 750 | 6.285 | 0.958 | 830.734 | 856.696 | 0.997 | 58.421 |
| SC-F46 | 647 | 5.052 | 0.892 | 749.07 | 746.15 | 0.997 | 51.338 |
| SC-F47 | 583 | 4.69 | 0.882 | 651.043 | 666.471 | 0.998 | 41.272 |
| SC-F48 | 751 | 6.124 | 0.948 | 874.367 | 882.551 | 0.997 | 64.568 |
| SC-F49 | 619 | 5.343 | 0.916 | 718.359 | 709.29 | 0.997 | 49.021 |
| SC-F50 | 165 | 3.521 | 0.851 | 221.438 | 214.642 | 0.999 | 17.154 |
| SC-F51 | 279 | 3.569 | 0.833 | 323.25 | 326.376 | 0.999 | 23.356 |
| SC-F52 | 671 | 5.733 | 0.944 | 813.235 | 804.738 | 0.997 | 50.107 |
| SC-F53 | 509 | 5.438 | 0.935 | 600.969 | 596.349 | 0.998 | 44.546 |
| SC-F54 | 559 | 4.69 | 0.874 | 746 | 714.997 | 0.997 | 44.717 |
| SC-F55 | 630 | 5.341 | 0.914 | 777.128 | 761.63 | 0.997 | 47.141 |
| SC-F56 | 609 | 5.849 | 0.957 | 764.013 | 766.018 | 0.997 | 49.672 |
| SC-F57 | 693 | 6.294 | 0.966 | 745.863 | 776.44 | 0.998 | 51.682 |
| SC-F58 | 691 | 5.659 | 0.943 | 852.284 | 813.355 | 0.997 | 59.947 |
| SC-F59 | 591 | 5.085 | 0.908 | 687.632 | 708.672 | 0.997 | 50.41 |
| SC-F60 | 752 | 5.467 | 0.911 | 913 | 887.436 | 0.997 | 57.413 |
| SC-F61 | 581 | 5.23 | 0.919 | 714.208 | 693.238 | 0.997 | 48.907 |
| SC-F62 | 642 | 5.485 | 0.923 | 700.593 | 717.625 | 0.998 | 51.032 |
| SC-F63 | 537 | 5.132 | 0.908 | 631.25 | 629.781 | 0.998 | 59.061 |
| SC-F64 | 593 | 4.9 | 0.881 | 700.361 | 698.258 | 0.997 | 48.221 |
| SC-F65 | 608 | 5.853 | 0.949 | 657.248 | 689.178 | 0.998 | 47.863 |
| SC-F66 | 626 | 5.748 | 0.956 | 683.095 | 703.355 | 0.998 | 46.5 |
| SC-F67 | 631 | 5.478 | 0.941 | 727.632 | 745.46 | 0.997 | 47.681 |
| SC-F68 | 764 | 6.474 | 0.972 | 839.375 | 859.741 | 0.997 | 58.831 |
| SC-F69 | 660 | 5.219 | 0.895 | 762.929 | 758.874 | 0.997 | 47.999 |
| SC-F70 | 477 | 5.258 | 0.918 | 565.393 | 558.287 | 0.998 | 40.162 |
| SC-F71 | 336 | 4.094 | 0.86 | 421.213 | 416.645 | 0.998 | 36.47 |
| SC-F72 | 625 | 5.301 | 0.926 | 688.629 | 718.822 | 0.997 | 47.38 |
| SC-F73 | 663 | 5.989 | 0.961 | 702.6 | 729.58 | 0.998 | 51.397 |
| SC-F74 | 619 | 5.794 | 0.951 | 765.81 | 713.991 | 0.997 | 50.801 |
| SC-F75 | 600 | 5.055 | 0.895 | 680.786 | 684.705 | 0.998 | 45.13 |
| SC-F76 | 512 | 5.825 | 0.96 | 602.833 | 601.233 | 0.998 | 39.689 |
| SC-F77 | 486 | 5.439 | 0.942 | 553.407 | 574.112 | 0.998 | 37.46 |
| LC-O1 | 754 | 5.456 | 0.936 | 945.801 | 1074.298 | 0.994 | 97.406 |
| LC-O2 | 545 | 5.645 | 0.955 | 631.143 | 678.787 | 0.997 | 60.877 |
| LC-O3 | 997 | 4.828 | 0.855 | 1426.767 | 1442.689 | 0.992 | 104.801 |
| LC-O4 | 423 | 4.625 | 0.901 | 546.092 | 565.135 | 0.997 | 66.172 |
| LC-O5 | 471 | 5.109 | 0.936 | 614.929 | 680.813 | 0.997 | 57.801 |
| LC-O6 | 417 | 4.899 | 0.921 | 493.901 | 519.78 | 0.998 | 56.113 |
| LC-O7 | 474 | 4.787 | 0.889 | 561.5 | 596.069 | 0.997 | 74.958 |
| LC-O8 | 412 | 4.609 | 0.906 | 450.537 | 475.534 | 0.998 | 59.006 |
| LC-O9 | 471 | 4.811 | 0.886 | 558.763 | 587.182 | 0.997 | 62.77 |
| LC-O10 | 404 | 4.657 | 0.898 | 491.803 | 507.496 | 0.998 | 59.07 |
| LC-O11 | 764 | 5.68 | 0.938 | 1078.711 | 1178.724 | 0.994 | 106.232 |
| LC-O12 | 784 | 4.927 | 0.893 | 1173.821 | 1147.844 | 0.994 | 105.363 |
| LC-O13 | 1652 | 6.098 | 0.927 | 1854.311 | 1989.817 | 0.991 | 138.527 |
| LC-O14 | 750 | 5.291 | 0.935 | 1130.395 | 1157.273 | 0.994 | 108.22 |
| LC-O15 | 884 | 4.674 | 0.831 | 1209.517 | 1332.914 | 0.993 | 108.007 |
| LC-O16 | 908 | 4.949 | 0.899 | 1158.133 | 1282.476 | 0.993 | 143.178 |
| LC-O17 | 748 | 4.874 | 0.881 | 1114.961 | 1183.711 | 0.993 | 96.219 |
| LC-O18 | 738 | 4.199 | 0.785 | 1072.701 | 1163.234 | 0.994 | 100.864 |
| LC-O19 | 1226 | 5.825 | 0.939 | 1425.985 | 1586.216 | 0.992 | 144.944 |
| LC-O20 | 820 | 4.904 | 0.886 | 1133.971 | 1167.03 | 0.994 | 119.371 |
| LC-O21 | 1384 | 5.425 | 0.869 | 1603.19 | 1708.125 | 0.992 | 121.849 |
| LC-O22 | 1011 | 4.934 | 0.894 | 1495.335 | 1605.743 | 0.991 | 134.656 |
| LC-O23 | 791 | 5.227 | 0.92 | 1163.28 | 1226.06 | 0.993 | 138.553 |
| LC-O24 | 766 | 4.933 | 0.895 | 1087.828 | 1205.036 | 0.994 | 89.274 |
| LC-O25 | 1883 | 6.946 | 0.957 | 2193.029 | 2233.538 | 0.99 | 154.898 |
| LC-O26 | 733 | 4.837 | 0.867 | 1023.878 | 1077.814 | 0.994 | 86.525 |
| LC-O27 | 839 | 5.336 | 0.934 | 1016.336 | 1152.852 | 0.994 | 115.305 |
| LC-O28 | 833 | 5.008 | 0.911 | 1224.506 | 1350.537 | 0.993 | 122.468 |
| LC-O29 | 720 | 5.305 | 0.94 | 1060.691 | 1138.431 | 0.994 | 97.573 |
| LC-O30 | 759 | 5.278 | 0.93 | 1114.474 | 1199.074 | 0.993 | 101.156 |
| LC-O31 | 1323 | 6.113 | 0.956 | 1600.071 | 1779.759 | 0.991 | 154.031 |
| LC-O32 | 702 | 4.647 | 0.875 | 969.363 | 1015.385 | 0.995 | 87.513 |
| LC-O33 | 898 | 4.746 | 0.86 | 1308.3 | 1388.382 | 0.992 | 99.659 |
| LC-O34 | 1140 | 5.259 | 0.91 | 1631.455 | 1734.79 | 0.99 | 152.704 |
| LC-O35 | 1321 | 5.058 | 0.862 | 1854.004 | 1971.555 | 0.989 | 159.172 |
| LC-O36 | 1085 | 5.331 | 0.934 | 1644.836 | 1829.995 | 0.99 | 147.397 |
| LC-O37 | 2055 | 6.582 | 0.953 | 2476.166 | 2698.192 | 0.986 | 173.821 |
| SC-O38 | 911 | 3.063 | 0.55 | 1302.005 | 1483.578 | 0.992 | 128.071 |
| SC-O39 | 1081 | 3.666 | 0.649 | 1798.155 | 1960.205 | 0.989 | 150.523 |
| SC-O40 | 2025 | 6.803 | 0.955 | 2106.514 | 2184.847 | 0.993 | 292.179 |
| SC-O41 | 1191 | 4.873 | 0.837 | 1762.332 | 1820.738 | 0.99 | 163.755 |
| SC-O42 | 943 | 4.022 | 0.741 | 1469.506 | 1612.512 | 0.991 | 135.174 |
| SC-O43 | 1438 | 4.937 | 0.862 | 2013.162 | 2118.361 | 0.988 | 161.735 |
| SC-O44 | 987 | 4.615 | 0.841 | 1611.94 | 1701.497 | 0.99 | 132.479 |
| SC-O45 | 959 | 4.337 | 0.83 | 1234.275 | 1389.842 | 0.992 | 112.136 |
| SC-O46 | 2560 | 7.063 | 0.952 | 2872.711 | 2918.899 | 0.988 | 331.551 |
| SC-O47 | 937 | 4.069 | 0.781 | 1391.366 | 1421.859 | 0.992 | 158.576 |
| SC-O48 | 1092 | 4.833 | 0.834 | 1509.029 | 1583.801 | 0.991 | 138.25 |
| SC-O49 | 2139 | 7.263 | 0.938 | 2415.734 | 2557.213 | 0.989 | 180.795 |
| SC-O50 | 929 | 3.867 | 0.732 | 1138.141 | 1270.192 | 0.993 | 117.634 |
| SC-O51 | 1007 | 4.512 | 0.815 | 1532.627 | 1542.435 | 0.991 | 128.117 |
| SC-O52 | 962 | 4.654 | 0.879 | 1507.741 | 1531.121 | 0.991 | 117.286 |
| SC-O53 | 851 | 4.093 | 0.802 | 1171.508 | 1311.363 | 0.993 | 105.859 |
| SC-O54 | 765 | 5.441 | 0.928 | 941.86 | 1049.031 | 0.995 | 90.43 |
| SC-O55 | 1427 | 5.474 | 0.913 | 2081.5 | 2131.336 | 0.988 | 151.261 |
| SC-O56 | 832 | 3.741 | 0.711 | 1278.959 | 1356.884 | 0.992 | 107.913 |
| SC-O57 | 920 | 4.352 | 0.85 | 1316.442 | 1343.622 | 0.992 | 96.83 |
| SC-O58 | 1226 | 4.451 | 0.805 | 1530.234 | 1721.029 | 0.99 | 144.594 |
| SC-O59 | 1011 | 5.165 | 0.906 | 1266.498 | 1424.935 | 0.992 | 140.664 |
| SC-O60 | 669 | 5.161 | 0.929 | 975.01 | 1014.452 | 0.995 | 96.446 |
| SC-O61 | 1499 | 5.533 | 0.892 | 2046.136 | 2095.966 | 0.989 | 141.294 |
| SC-O62 | 489 | 3.527 | 0.709 | 690.375 | 710.397 | 0.996 | 81.244 |
| SC-O63 | 828 | 4.845 | 0.894 | 1291.854 | 1385.957 | 0.992 | 125.213 |
| SC-O64 | 531 | 4.533 | 0.887 | 730 | 794.853 | 0.996 | 84.552 |
| SC-O65 | 929 | 4.677 | 0.869 | 1257.13 | 1405.095 | 0.992 | 143.157 |
| SC-O66 | 668 | 4.424 | 0.829 | 863 | 948.713 | 0.995 | 103.146 |
| SC-O67 | 710 | 3.513 | 0.666 | 1066.483 | 1110.624 | 0.994 | 123.332 |
| SC-O68 | 604 | 3.901 | 0.719 | 799.82 | 839.967 | 0.996 | 90.577 |
| SC-O69 | 704 | 4.344 | 0.813 | 997.201 | 1087.883 | 0.994 | 109.371 |
| SC-O70 | 556 | 4.623 | 0.854 | 674.456 | 765.242 | 0.996 | 80.91 |
| SC-O71 | 1084 | 5.067 | 0.885 | 1698.043 | 1786.444 | 0.99 | 147.98 |
| SC-O72 | 586 | 4.734 | 0.869 | 813.043 | 842.26 | 0.996 | 73.109 |
| SC-O73 | 1213 | 5.111 | 0.885 | 1670.067 | 1736.358 | 0.99 | 126.893 |
| SC-O74 | 431 | 2.336 | 0.491 | 639.154 | 645.316 | 0.997 | 76.467 |
| SC-O75 | 533 | 3.986 | 0.807 | 781.929 | 800.229 | 0.996 | 79.868 |
| SC-O76 | 496 | 5.04 | 0.926 | 609.624 | 666.539 | 0.997 | 73.954 |
| SC-O77 | 478 | 4.521 | 0.898 | 594.753 | 668.07 | 0.997 | 74.468 |
| SC-O78 | 544 | 5.317 | 0.914 | 607.917 | 643.012 | 0.998 | 68.556 |
| SC-O79 | 599 | 3.978 | 0.789 | 750.964 | 787.038 | 0.996 | 93.066 |
| SC-O80 | 468 | 4.586 | 0.899 | 574.68 | 604.388 | 0.997 | 70.345 |
| SC-O81 | 489 | 3.914 | 0.849 | 617.485 | 665.242 | 0.997 | 87.955 |
| SC-O82 | 650 | 3.352 | 0.73 | 990.084 | 1075.564 | 0.994 | 132.873 |
| SC-O83 | 2050 | 5.616 | 0.887 | 2711.736 | 2796.822 | 0.985 | 255.85 |
| SC-O84 | 895 | 4.861 | 0.924 | 1323.784 | 1429.665 | 0.992 | 125.088 |
| SC-O85 | 1982 | 6.055 | 0.895 | 2261.139 | 2406.797 | 0.989 | 193.257 |

Supplementary Table 3 Benjamini & Hochberg (BH) correction for the LefSe results of gut microbiota.

| **Gut microbiota** | **p value** | **Adj p value (BH)** |
| --- | --- | --- |
| p__Firmicutes_c__Clostridia_o__Lachnospirales_f__Lachnospiraceae_g__*Roseburia*_s__*Roseburia_intestinalis* | 7.17E-12 | 4.30E-10 |
| p__Firmicutes_c__Bacilli_o__Erysipelotrichales_f__Erysipelotrichaceae_g__*Faecalibaculum* | 3.55E-11 | 7.10E-10 |
| p__Firmicutes_c__Bacilli_o__Erysipelotrichales_f__Erysipelotrichaceae_g__*Faecalibaculum*_s__*Faecalibaculum_rodentium* | 3.55E-11 | 7.10E-10 |
| p__Firmicutes_c__Clostridia_o__Lachnospirales_f__Lachnospiraceae_g__*Roseburia* | 8.27E-10 | 1.16E-08 |
| p__Firmicutes_c__Clostridia_o__Oscillospirales_f__Ruminococcaceae_g__*Subdoligranulum* | 9.63E-10 | 1.16E-08 |
| p__unidentified_Bacteria_c__Gammaproteobacteria | 2.43E-09 | 2.43E-08 |
| p__unidentified_Bacteria_c__Gammaproteobacteria_o__Enterobacterales_f__Succinivibrionaceae | 1.05E-08 | 7.88E-08 |
| p__unidentified_Bacteria_c__Gammaproteobacteria_o__Enterobacterales | 1.05E-08 | 7.88E-08 |
| p__Firmicutes_c__Clostridia_o__Lachnospirales_f__Lachnospiraceae_g__*Coprococcus* | 4.03E-08 | 2.69E-07 |
| p__Firmicutes_c__Clostridia_o__Clostridiales_f__Clostridiaceae_g__*Sarcina* | 7.22E-08 | 4.25E-07 |
| p__Firmicutes_c__Clostridia_o__Lachnospirales_f__Lachnospiraceae_g__*Coprococcus*_s__*Coprococcus_eutactus* | 7.79E-08 | 4.25E-07 |
| p__Spirochaetota_c__Spirochaetia_o__Spirochaetales_f__Spirochaetaceae_g__*Treponema*_s__*Treponema_brennaborense* | 2.36E-07 | 1.18E-06 |
| p__Firmicutes_c__Clostridia_o__Lachnospirales_f__Lachnospiraceae_g__*Blautia* | 2.49E-06 | 1.15E-05 |
| p__Bacteroidota_c__Bacteroidia_o__Bacteroidales_f__Muribaculaceae | 1.43E-05 | 6.13E-05 |
| p__Firmicutes_c__Clostridia_o__Lachnospirales_f__Lachnospiraceae_g___*Ruminococcus*__gnavus_group | 1.89E-05 | 7.56E-05 |
| p__Desulfobacterota_c__Desulfovibrionia_o__Desulfovibrionales_f__Desulfovibrionaceae_g__*Desulfovibrio* | 7.19E-05 | 2.70E-04 |
| p__Firmicutes_c__Negativicutes_o__Veillonellales_Selenomonadales_f__Veillonellaceae_g__*Megasphaera*_s__*Megasphaera_elsdenii* | 0.0001 | 0.0003 |
| p__Desulfobacterota_c__Desulfovibrionia_o__Desulfovibrionales_f__Desulfovibrionaceae_g__*Desulfovibrio*_s__*Desulfovibrio_piger* | 0.0001 | 0.0004 |
| p__Actinobacteriota_c__Coriobacteriia_o__Coriobacteriales_f__Coriobacteriaceae_g__*Collinsella*_s__*Collinsella_aerofaciens* | 0.0002 | 0.0006 |
| p__Firmicutes_c__Negativicutes_o__Acidaminococcales_f__Acidaminococcaceae | 0.0002 | 0.0007 |
| p__Firmicutes_c__Negativicutes_o__Acidaminococcales | 0.0002 | 0.0007 |
| p__Firmicutes_c__Clostridia_o__Clostridiales_f__Clostridiaceae | 0.0003 | 0.0009 |
| p__Firmicutes_c__Clostridia_o__Clostridiales | 0.0003 | 0.0009 |
| p__Firmicutes_c__Clostridia_o__Clostridiales_f__Clostridiaceae_g__*Clostridium*_sensu_stricto_1_s__*Clostridium_butyricum* | 0.0004 | 0.0010 |
| p__Desulfobacterota_c__Desulfovibrionia | 0.0007 | 0.0016 |
| p__Desulfobacterota_c__Desulfovibrionia_o__Desulfovibrionales_f__Desulfovibrionaceae | 0.0007 | 0.0016 |
| p__Desulfobacterota_c__Desulfovibrionia_o__Desulfovibrionales | 0.0007 | 0.0016 |
| p__Actinobacteria_c__unidentified_Actinobacteria_o__Bifidobacteriales_f__Bifidobacteriaceae_g__*Bifidobacterium*_s__*Bifidobacterium*_animalis | 0.0011 | 0.0023 |
| p__Actinobacteriota_c__Coriobacteriia_o__Coriobacteriales_f__Coriobacteriaceae_g__*Collinsella* | 0.0016 | 0.0034 |
| p__Desulfobacterota | 0.0018 | 0.0035 |
| p__Actinobacteriota_c__Coriobacteriia_o__Coriobacteriales_f__Coriobacteriaceae | 0.0026 | 0.0050 |
| p__Firmicutes_c__Clostridia_o__Lachnospirales_f__Lachnospiraceae_g__Lachnospiraceae_NK4A136_group_s__Lachnospiraceae_bacterium_GAM79 | 0.0032 | 0.0058 |
| p__Cyanobacteria_c__Cyanobacteriia_o__Chloroplast_f__unidentified_Chloroplast | 0.0034 | 0.0058 |
| p__Cyanobacteria_c__Cyanobacteriia_o__Chloroplast | 0.0034 | 0.0058 |
| p__Cyanobacteria_c__Cyanobacteriia_o__Chloroplast_f__unidentified_Chloroplast_g__unidentified_Chloroplast | 0.0034 | 0.0058 |
| p__Bacteroidota_c__Bacteroidia_o__Bacteroidales_f__Rikenellaceae | 0.0041 | 0.0069 |
| p__Actinobacteriota | 0.0054 | 0.0088 |
| p__Actinobacteriota_c__Coriobacteriia_o__Coriobacteriales | 0.0064 | 0.0098 |
| p__Actinobacteriota_c__Coriobacteriia | 0.0064 | 0.0098 |
| p__Firmicutes_c__Bacilli_o__Erysipelotrichales_f__Erysipelotrichaceae | 0.0070 | 0.0105 |
| p__Actinobacteria_c__unidentified_Actinobacteria_o__Corynebacteriales_f__Corynebacteriaceae | 0.0082 | 0.0115 |
| p__Actinobacteria_c__unidentified_Actinobacteria_o__Corynebacteriales_f__Corynebacteriaceae_g__*Corynebacterium* | 0.0082 | 0.0115 |
| p__Bacteroidota_c__Bacteroidia_o__Bacteroidales_f__Rikenellaceae_g__*Alistipes* | 0.0083 | 0.0115 |
| p__Fusobacteriota_c__Fusobacteriia | 0.0092 | 0.0117 |
| p__Fusobacteriota_c__Fusobacteriia_o__Fusobacteriales | 0.0092 | 0.0117 |
| p__Fusobacteriota | 0.0092 | 0.0117 |
| p__Fusobacteriota_c__Fusobacteriia_o__Fusobacteriales_f__Fusobacteriaceae_g__*Fusobacterium* | 0.0092 | 0.0117 |
| p__Fusobacteriota_c__Fusobacteriia_o__Fusobacteriales_f__Fusobacteriaceae | 0.0100 | 0.0125 |
| p__Firmicutes_c__Clostridia_o__Clostridiales_f__Clostridiaceae_g__*Clostridium*_sensu_stricto_1 | 0.0110 | 0.0133 |
| p__Cyanobacteria_c__Cyanobacteriia | 0.0110 | 0.0133 |
| p__Actinobacteria_c__unidentified_Actinobacteria_o__Corynebacteriales | 0.0127 | 0.0149 |
| p__Spirochaetota_c__Spirochaetia_o__Spirochaetales_f__Spirochaetaceae_g__*Sphaerochaeta*_s__*Sphaerochaeta*_sp | 0.0134 | 0.0155 |
| p__Bacteroidota_c__Bacteroidia_o__Bacteroidales_f__Bacteroidaceae_g__*Bacteroides*_s__*Bacteroides_fragilis* | 0.0199 | 0.0225 |
| p__Firmicutes_c__Clostridia_o__Oscillospirales_f__Oscillospiraceae | 0.0204 | 0.0227 |
| p__Fusobacteriota_c__Fusobacteriia_o__Fusobacteriales_f__Fusobacteriaceae_g__*Fusobacterium*_s__*Fusobacterium_mortiferum* | 0.0231 | 0.0252 |
| p__Cyanobacteria_c__Vampirivibrionia_o__Gastranaerophilales | 0.0280 | 0.0300 |
| p__Cyanobacteria_c__Vampirivibrionia | 0.0287 | 0.0302 |
| p__Bacteroidota_c__Bacteroidia_o__Bacteroidales_f__Bacteroidaceae_g__*Bacteroides*_s__*Bacteroides_cellulosilyticus* | 0.0312 | 0.0323 |
| p__Firmicutes_c__Clostridia_o__Lachnospirales | 0.0383 | 0.0383 |
| p__Firmicutes_c__Clostridia_o__Lachnospirales_f__Lachnospiraceae | 0.0383 | 0.0383 |

Supplementary Table 4 Benjamini & Hochberg (BH) correction for the LefSe results of oral microbiota.

| **Oral microbiota** | **p value** | **Adj p value (BH)** |
| --- | --- | --- |
| p__Bacteroidota.c__Bacteroidia | 2.20E-10 | 5.83E-09 |
| p__Bacteroidota | 2.20E-10 | 5.83E-09 |
| p__Bacteroidota.c__Bacteroidia.o__Flavobacteriales.f__Flavobacteriaceae | 1.03E-08 | 1.82E-07 |
| p__Bacteroidota.c__Bacteroidia.o__Flavobacteriales.f__Flavobacteriaceae.g__*Capnocytophaga* | 1.64E-08 | 2.17E-07 |
| p__Bacteroidota.c__Bacteroidia.o__Bacteroidales.f__Porphyromonadaceae | 2.35E-08 | 2.28E-07 |
| p__Bacteroidota.c__Bacteroidia.o__Bacteroidales.f__Porphyromonadaceae.g__*Porphyromonas* | 2.60E-08 | 2.28E-07 |
| p__Bacteroidota.c__Bacteroidia.o__Bacteroidales | 3.03E-08 | 2.28E-07 |
| p__Fusobacteriota.c__Fusobacteriia | 4.31E-08 | 2.28E-07 |
| p__Fusobacteriota.c__Fusobacteriia.o__Fusobacteriales | 4.31E-08 | 2.28E-07 |
| p__Fusobacteriota | 4.31E-08 | 2.28E-07 |
| p__Fusobacteriota.c__Fusobacteriia.o__Fusobacteriales.f__Fusobacteriaceae.g__*Fusobacterium.*s__*Fusobacterium_nucleatum* | 1.15E-07 | 5.54E-07 |
| p__Bacteroidota.c__Bacteroidia.o__Flavobacteriales | 1.40E-07 | 6.18E-07 |
| p__Fusobacteriota.c__Fusobacteriia.o__Fusobacteriales.f__Fusobacteriaceae | 1.87E-07 | 7.62E-07 |
| p__Fusobacteriota.c__Fusobacteriia.o__Fusobacteriales.f__Fusobacteriaceae.g__*Fusobacterium* | 2.06E-07 | 7.80E-07 |
| p__Bacteroidota.c__Bacteroidia.o__Flavobacteriales.f__Flavobacteriaceae.g__*Capnocytophaga.*s__*Capnocytophaga_ochracea* | 3.00E-07 | 1.06E-06 |
| p__Proteobacteria | 4.17E-07 | 1.38E-06 |
| p__Proteobacteria.c__Gammaproteobacteria | 7.58E-07 | 2.36E-06 |
| p__Fusobacteriota.c__Fusobacteriia.o__Fusobacteriales.f__Leptotrichiaceae | 8.68E-07 | 2.56E-06 |
| p__Actinobacteria.c__unidentified_Actinobacteria.o__Micrococcales.f__Micrococcaceae.g__*Rothia* | 1.46E-05 | 4.07E-05 |
| p__Actinobacteria.c__unidentified_Actinobacteria.o__Micrococcales.f__Micrococcaceae.g__*Rothia.*s__*Rothia_aeria* | 1.72E-05 | 4.56E-05 |
| p__Bacteroidota.c__Bacteroidia.o__Bacteroidales.f__Prevotellaceae | 2.14E-05 | 5.40E-05 |
| p__Proteobacteria.c__Gammaproteobacteria.o__Pseudomonadales.f__Pseudomonadaceae | 2.76E-05 | 6.08E-05 |
| p__Actinobacteria | 2.87E-05 | 6.08E-05 |
| p__Proteobacteria.c__Gammaproteobacteria.o__Pseudomonadales.f__Pseudomonadaceae.g__*Pseudomonas* | 2.87E-05 | 6.08E-05 |
| p__Actinobacteria.c__unidentified_Actinobacteria | 2.87E-05 | 6.08E-05 |
| p__Fusobacteriota.c__Fusobacteriia.o__Fusobacteriales.f__Leptotrichiaceae.g__*Leptotrichia* | 3.10E-05 | 6.32E-05 |
| p__Proteobacteria.c__Gammaproteobacteria.o__Burkholderiales.f__Neisseriaceae | 4.06E-05 | 7.97E-05 |
| p__Proteobacteria.c__Gammaproteobacteria.o__Burkholderiales.f__Neisseriaceae.g__*Neisseria.*s__*Neisseria_oralis* | 5.11E-05 | 9.67E-05 |
| p__Proteobacteria.c__Gammaproteobacteria.o__Burkholderiales.f__Neisseriaceae.g__*Neisseria* | 8.00E-05 | 1.46E-04 |
| p__Actinobacteria.c__unidentified_Actinobacteria.o__Actinomycetales.f__Actinomycetaceae | 9.97E-05 | 1.70E-04 |
| p__Actinobacteria.c__unidentified_Actinobacteria.o__Actinomycetales | 9.97E-05 | 1.70E-04 |
| p__Actinobacteria.c__unidentified_Actinobacteria.o__Actinomycetales.f__Actinomycetaceae.g__*Actinomyces* | 1.48E-04 | 2.46E-04 |
| p__Proteobacteria.c__Gammaproteobacteria.o__Burkholderiales.f__Burkholderiaceae.g__*Ralstonia* | 2.35E-04 | 3.77E-04 |
| p__Proteobacteria.c__Gammaproteobacteria.o__Burkholderiales.f__Burkholderiaceae.g__*Ralstonia.*s__*Ralstonia_pickettii* | 2.43E-04 | 3.79E-04 |
| p__Proteobacteria.c__Gammaproteobacteria.o__Burkholderiales.f__Burkholderiaceae.g__*Lautropia* | 3.20E-04 | 4.84E-04 |
| p__Actinobacteria.c__unidentified_Actinobacteria.o__Micrococcales | 7.81E-04 | 0.0012 |
| p__Proteobacteria.c__Gammaproteobacteria.o__Pseudomonadales | 0.0011 | 0.0015 |
| p__Proteobacteria.c__Gammaproteobacteria.o__Enterobacterales | 0.0023 | 0.0031 |
| p__Firmicutes.c__Bacilli.o__Staphylococcales.f__Gemellaceae.g__*Gemella* | 0.0024 | 0.0031 |
| p__Firmicutes.c__Bacilli.o__Staphylococcales.f__Gemellaceae | 0.0024 | 0.0031 |
| p__Actinobacteria.c__unidentified_Actinobacteria.o__Micrococcales.f__Micrococcaceae | 0.0024 | 0.0031 |
| p__Proteobacteria.c__Gammaproteobacteria.o__Enterobacterales.f__Pasteurellaceae | 0.0027 | 0.0034 |
| p__Actinobacteria.c__unidentified_Actinobacteria.o__Corynebacteriales | 0.0037 | 0.0046 |
| p__Bacteroidota.c__Bacteroidia.o__Bacteroidales.f__Porphyromonadaceae.g__*Porphyromonas.*s__*Porphyromonas_gingivalis* | 0.0062 | 0.0074 |
| p__Firmicutes.c__Negativicutes.o__Veillonellales_Selenomonadales.f__Veillonellaceae.g__*Veillonella.*s_*_Veillonella_parvula* | 0.0067 | 0.0079 |
| p__Actinobacteria.c__unidentified_Actinobacteria.o__Corynebacteriales.f__Corynebacteriaceae.g__*Corynebacterium* | 0.0070 | 0.0081 |
| p__Actinobacteria.c__unidentified_Actinobacteria.o__Corynebacteriales.f__Corynebacteriaceae | 0.0072 | 0.0082 |
| p__Firmicutes.c__Negativicutes.o__Veillonellales_Selenomonadales.f__Veillonellaceae.g__*Veillonella* | 0.0080 | 0.0089 |
| p__Firmicutes.c__Negativicutes.o__Veillonellales_Selenomonadales.f__Veillonellaceae | 0.0163 | 0.0176 |
| p__Proteobacteria.c__Gammaproteobacteria.o__Burkholderiales | 0.0244 | 0.0258 |
| p__Firmicutes.c__Negativicutes.o__Veillonellales_Selenomonadales | 0.0261 | 0.0271 |
| p__Bacteroidota.c__Bacteroidia.o__Flavobacteriales.f__Weeksellaceae | 0.0306 | 0.0311 |
| p__Firmicutes.c__Negativicutes | 0.0312 | 0.0312 |

## Supplementary Figures


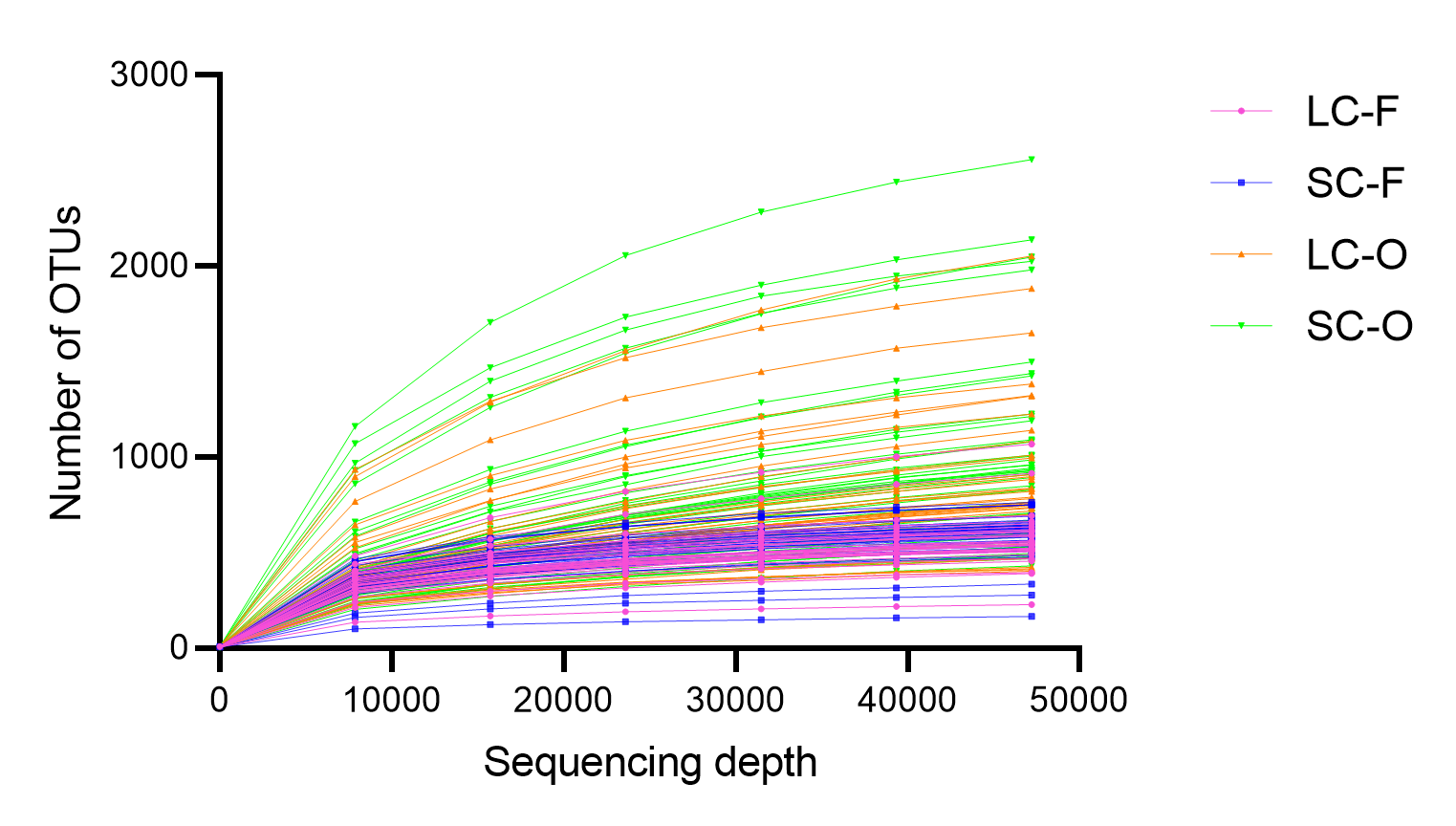


**Supplementary Figure 1.** Rarefaction curve indicated the observed OTUs (mean ± SD) against sequencing depth in each group**.** *LC-F* (long-term captivity-fecal), *SC-F* (short-term captivity-fecal), *LC-O* (long-term captivity-oral), *SC-O* (short-term captivity-oral)


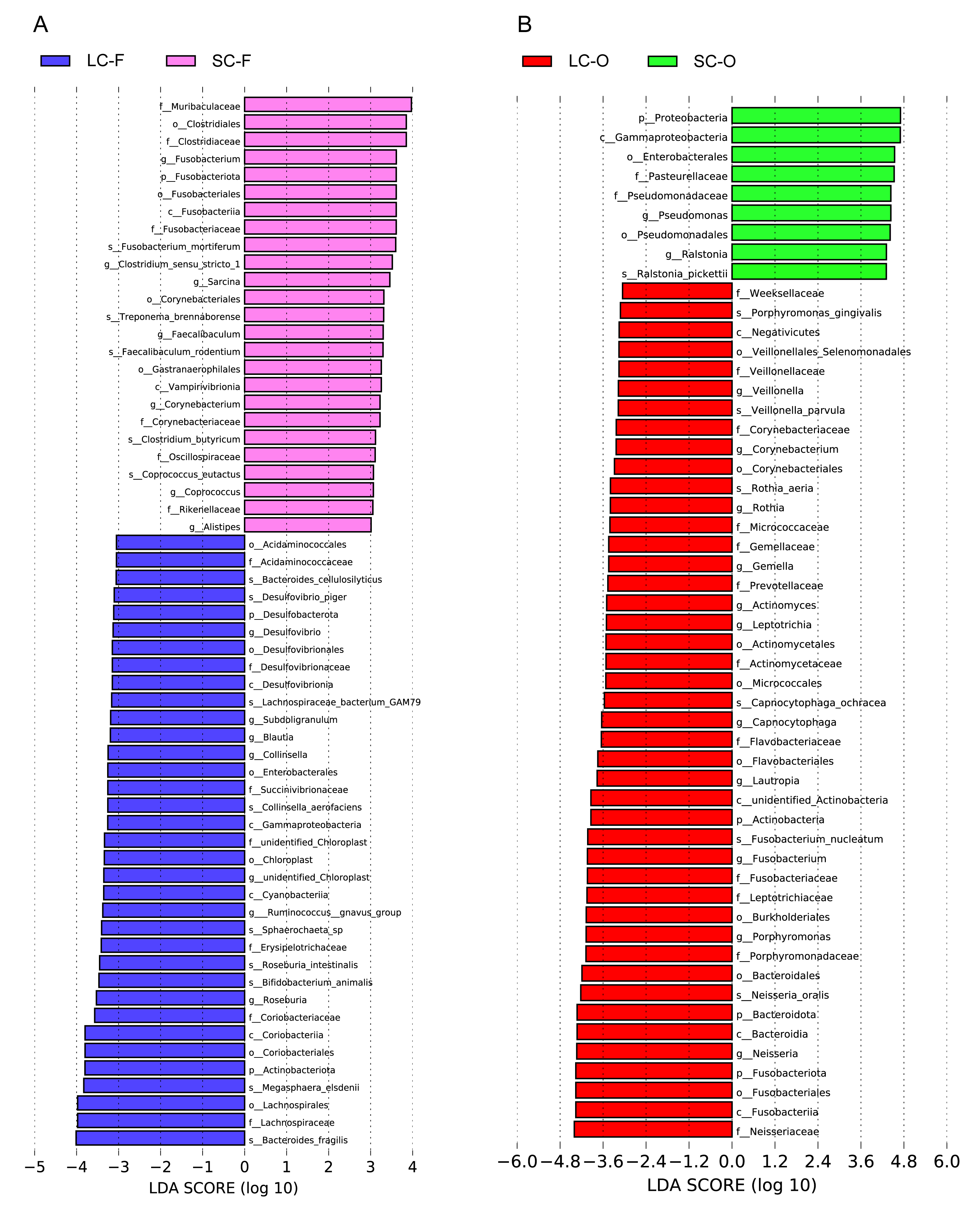


**Supplementary Figure 2.** Species with significant difference that have an LDA score ≥4.0.
